# LINC01088 prevents ferroptosis in glioblastoma by enhancing SLC7A11 via HLTF/USP7 axis

**DOI:** 10.1002/ctm2.70257

**Published:** 2025-02-25

**Authors:** Yujie Zhou, Zhen Zhao, Cheng Jiang, Chuansheng Nie, Dongdong Xiao, Zhipeng Wu, Hao Yu, Jianglin Zheng, Xuan Wang, Xiaobing Jiang

**Affiliations:** ^1^ Department of Neurosurgery, Union Hospital, Tongji Medical College Huazhong University of Science and Technology Wuhan China; ^2^ Department of Neurosurgery Weifang People's Hospital Weifang Shandong China

**Keywords:** cystine transporter, ferroptosis, glioblastoma, LncRNA, transcription factor

## Abstract

**Background:**

Glioblastoma multiforme (GBM)is a highly aggressive malignancy of the central nervous system characterized by poor survival rates. Ferroptosis, an iron‐dependent cell death pathway, is a promising therapeutic target for GBM. However, current treatments targeting cell death pathways have not yielded expected results. Long noncoding RNAs (lncRNAs) have been implicated in tumour proliferation, however, their role in ferroptosis in GBM remains underexplored. This study investigated the interplay between the lncRNA LINC01088 and ferroptosis in GBM to identify novel therapeutic strategies.

**Methods:**

We conducted gain‐ and loss‐of‐function studies to assess the impact of LINC01088 on GBM tumourigenesis and ferroptosis both in vitro and in vivo. Bioinformatics, dual‐luciferase reporter assays, chromatin immunoprecipitation, RNA pulldown, mass spectrometry, RNA immunoprecipitation (RIP), and transcriptome sequencing were utilized to elucidate the mechanisms underlying LINC01088 expression and its downstream effects on ferroptosis.

**Results:**

The transcription factor specificity protein 1 (SP1) was identified as the promoter of LINC01088 transcription, which facilitated GBM progression. LINC01088 was found to inhibit ferroptosis and promote malignancy. Mechanistically, LINC01088 stabilized HLTF by enhancing its interaction with USP7 and preventing ubiquitin‐mediated degradation. The stabilization of HLTF led to the upregulation of SLC7A11, which inhibits ferroptosis in GBM. Rescue experiments confirmed that altering HLTF levels reversed the ferroptotic phenotypes associated with LINC01088 modulation.

**Conclusion:**

This study revealed a novel SP1/LINC01088/HLTF/USP7/SLC7A11 axis that regulates ferroptosis in GBM, highlighting LINC01088 as a potential therapeutic target for ferroptosis‐dependent GBM treatment.

**Key points:**

- LINC01088 is transcriptionally upregulated by SP1.
- LINC01088 acts as a scaffold platform to bind USP7 and HLTF.
- USP7, as a deubiquitinating enzyme of HLTF, participates in inhibiting the ubiquitin‐proteasome degradation of HLTF.
- HLTF transcriptionally upregates the expression of downstream SLC7A11, and ferroptosis of GBM cells was inhibited.

## Graphical abstract

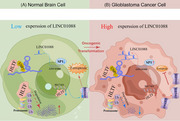

## 1 INTRODUCTION

Glioblastoma multiforme (GBM) is a primary intracranial tumour characterized by high invasiveness.^1^, ^2^, ^3^ Despite the availability of various treatment modalities, such as surgery, radiation therapy, and chemotherapy, the overall survival of patients remains approximately 15 months.^2^, ^3^ Numerous studies have sought to elucidate various cell death pathways in tumour cells, however significant theoretical breakthroughs in the regulation of cell death mechanisms have yet to be achieved.^4^, ^5^, ^6^, ^7^, ^8^, ^9^ Therefore, novel and effective treatment strategies for GBM urgently need to improve patient outcomes and survival prospects.

With the rapid development of high‐throughput sequencing technologies, long noncoding RNAs (lncRNAs) have attracted increasing attention in recent years.^10^, ^11^, ^12^, ^13^, ^14^, ^15^ Generally, lncRNAs are considered non‐protein‐coding RNAs with a minimum length of 200 nucleotides and play important roles in the development of various cancers. Therefore, the study of lncRNAs associated with tumours and their potential molecular mechanisms of action offers hope for identifying new GBM biomarkers and therapeutic targets.

Resistance to cell death is a crucial hallmark of tumours, it promotes tumour progression and leads to an inherent or acquired resistance to treatment in most patients.^16^ Therefore, understanding the mechanisms of underlying cell death resistance is essential for improving glioma patient outcomes. Ferroptosis is a unique and recently recognized mode of regulated cell death characterized by iron‐dependent lipid peroxidation.^17^, ^18^ Excess intracellular iron ions lead to glutathione (GSH) depletion, the accumulation of reactive oxygen species (ROS), and ultimately cell death.^19^ Chemotherapy‐resistant GBM cells and other tumour cells, particularly stromal and metastatic tumour cells, are relatively sensitive to ferroptosis induced by glutathione peroxidase‐4 (GPX4).^20^ Recent studies have demonstrated that targeting cell death pathways is a promising therapeutic strategy to prevent GBM progression.^17^, ^21^ For example, a combination of immunotherapy and cell death‐targeting drugs has been found to inhibit tumour growth in GBM mouse models.^22^ Unfortunately, these studies did not achieve the expected results and the regulatory mechanisms of ferroptosis in GBM remain poorly understood.

In this study, we used various bioinformatics approaches to identify the lncRNA, LINC01088, as a potential GBM regulator. Although LINC01088 has been found to promote or suppress tumours in other cancers,^23^, ^24^, ^25^, ^26^ its regulatory mechanism in GBM remains unclear, and its association with ferroptosis has not been investigated. Therefore, we focussed on exploring the tumour regulatory mechanisms of LINC01088 in GBM and its relationship with ferroptosis. LINC01088 was found to be significantly upregulated in GBM, primarily due to direct positive transcriptional regulation by the transcription factor 1(SP1). We found that LINC01088 promoted GBM cell proliferation, migration, and invasion and inhibited ferroptosis in GBM cells. Mechanistically, LINC01088, acting as a scaffold, upregulated the transcription of SLC7A11 by interacting with USP7/HLTF, thereby inhibiting ferroptosis in GBM cells. This study elucidates the role and detailed mechanism of LINC01088/HLTF/USP7/SLC7A11 in GBM ferroptosis, providing theoretical support for improving GBM treatments by promoting ferroptosis.

## 2 MATERIALS AND METHODS

### 2.1 Patients and tissue samples

Tumour tissue samples were collected from 30 patients with glioblastoma who underwent inpatient treatment at the Department of Neurosurgery, Affiliated Union Hospital, Tongji Medical College, Huazhong University of Science and Technology from September 2020 to September 2021. Postoperative pathological examination confirmed the diagnosis of glioblastoma in all cases. Brain tissue samples were collected from 30 non‐tumour patients (suffering from traumatic brain injury) to serve as experimental controls. Detailed patient information is provided in Table S1. None of the patients received anti‐cancer therapy prior to surgery. All the specimens were excised and immediately snap‐frozen in liquid nitrogen. The study was approved by the Ethics Committee of the Huazhong University of Science and Technology.

### 2.2 Cell culture and chemicals

All GBM cancer cell lines were purchased from the American Type Culture Collection (ATCC). The normal astrocyte cell line (HA1800) was obtained from the Science Cell Laboratory. The cell lines were routinely characterized by DNA fingerprinting, cell vitality detection, isozyme detection, and mycoplasma detection in our laboratory.^27^, ^28^, ^29^ The cell culture media were supplemented with 10% foetal bovine serum (FBS, GIBCO), 100 U/mL penicillin, 100 mg/mL streptomycin (Thermo Fisher Scientific), and 8 mg/L antibiotic tylosin tartrate against mycoplasma (Sigma‐Aldrich) at 37°C in an atmosphere of 5% CO_2_. All cells were cultured in Dulbecco's Modified Eagle Medium (DMEM). Cells were used within 6 months of culturing and were regularly tested for mycoplasma contamination to ensure that they were mycoplasma‐free. MedChemExpress (MCE) provided MG132 (HY‐13259), erastin (HY‐15763), and cycloheximide (CHX, HY‐12320). The interference plasmid was transfected into U87MG and U251 glioblastoma cells using Lipofectamine 3000 (Thermo Fisher Scientific). The specific sequences of shRNAs and siRNAs employed are detailed in Table S2.

### 2.3 Nuclear and cytoplasmic extraction, RNA extraction, and RT‐qPCR

Nuclear and cytoplasmic RNAs were separated using a nuclear and cytoplasmic RNA purification kit (AM1921, Thermo Fisher Scientific). Total RNA from tissues or cell lines were extracted using Trizol (Takara) based on the manufacturer's instructions and assessed via Nanodrop. By using Evo M‐MLV RT Kit (AG11728, Accurate Biotechnology Co., Ltd), 1 µg of total RNA was reverse 120 transcribed into cDNA, which was used for subsequent RT‐qPCR via SYBR Green premix Pro Taq HS qPCR Kit (AG11728, Accurate Biotechnology Co., Ltd). Each reaction was performed on the Bio‐Rad Real time PCR system (Bio‐Rad). The internal reference for normalization of RT‐qPCR results was GAPDH, and relative expression was calculated using the 2(‐ΔΔCT) method. The primers involved were listed in Table S3.

### 2.4 Cell viability, colony formation, and EdU assays

The cell viability was measured by the Cell Counting Kit‐8 (CCK8, BS350, Biosharp). Cell suspension was planted into 96‐well plates (3 × 10^3^ cells/well) with 100 µL of medium. Subsequently, 10 µL CCK8 solution was added to each well at the time points of 24, 48, 72, and 96h. After incubation of 2 h at 37°C absorbance at 450 nm of each well was measured. For colony formation assay, glioma cells were cultured in 6‐well plates with 1 × 10^3^ cells per well. After 2 weeks of incubation, the cells were washed thrice with PBS, fixed with 4% paraformaldehyde for 15 min, and stained with 0.1% crystal violet solution for 12 min. The size and number of colonies were observed. For EdU assays, glioma cells were cultured in 48‐well plates until 50%–80% confluent. Then, BeyoClick™ EdU Cell Proliferation Kit with Alexa Fluor 594 (C0078L, Beyotime) was used according to the manufacturer's instructions. The proportion of EdU‐positive cells were quantified under a fluorescence microscope (Nexcope NE930, Ningbo, China).

### 2.5 Cell migration, invasion, and cycle assays

The cell migration and invasion assays were performed using transwell chambers with or without Matrigel membranes. In brief, a total of 1 × 10^4^ starved glioma cells were added to the upper chamber with 200 µL of serum‐free medium, and the lower chamber was applied with 600 µL of complete medium containing 20% FBS. After incubation for 36 h, cells attached to the upper surface were removed with a cotton, while cells that migrated or invaded into the lower surface of the membrane were fixed with 4% paraformaldehyde for 15 min, and stained with 0.1% crystal violet solution for 12 min, and counted in five random fields of view under an objective lens. For cell cycle assays, cells were collected and fixed in 75% ice‐cold ethanol at 4°C overnight. Next day, the fixed cells were washed with PBS thrice and stained with propidium iodide (Beyotime). Lastly, cell cycle analysis was performed using flow cytometry, and the results were visualized via ModFit LT software.

### 2.6 Immunohistochemistry (IHC) and terminal deoxynucleotidyl transferase dUTP nick end labelling (TUNEL) assay

Tissue specimens from human or mouse were fixed with 4% paraformaldehyde, embedded in paraffin, sectioned with 6‐µm thickness, and immunostained with specific antibodies. The histological slides were observed under a light microscope (Leica). The percentage of positive cells was calculated. TUNEL staining was performed with In Situ Cell Death Detection Kit, POD (Roche) according to the manufacturer's protocol. Images were acquired with an Olympus FSX100 microscope (Olympus).

### 2.7 Fluorescence in situ hybridization (FISH) assay

A Cy3‐labelled LINC01088 FISH probe was purchased from RiboBio and FISH assays were carried out with a Ribo FISH kit (C10910, RiboBio) according to the manufacturer's instructions. For immunofluorescence staining assays, cells were fixed with 4% paraformaldehyde at room temperature for 15 min, permeated with 0.5% Triton X‐100 for 10 min, blocked with 5% BSA for 1 h, incubated with primary antibodies at 4°C overnight and then with corresponding Fluor‐labelled secondary antibodies (1:200, Thermo Fisher Scientific). Nuclei was stained via DAPI (C1002, Beyotime) was used to stain nuclei. Images were taken by fluorescence microscope (Nexcope NE930).

### 2.8 Transmission electron microscopy

In brief, glioma cells were seeded into T25 culture flask and were treated with Erastin or DMSO for 48 h. Then, cells are collected after centrifuge and the precipitation. The IEM fixative was added to cells and let the cell precipitation resuspended in the fixative, and then fixed at 4°C for preservation. Images were obtained through transmission electron microscope (Hitachi, HT7700).

### 2.9 Dual‐luciferase assay

In brief, cells seeded in 24‐well plates with 60–80% confluency were transfected with dual‐luciferase reporters. Forty‐eight hours after transfection, renilla and firefly luciferase activities in each well were detected by using HBLumi dual‐luciferase reporter assay kit (Hanbio) according to the manufacturer's instructions. Detailed sequences are listed in Tables S4 and S5.

### 2.10 Mass spectrometry and RNA‐sequencing

Mass spectrometry was performed by SpecAlly Life 159 Technology Co., Ltd, the results were shown on Tables S6 and S7. After si‐HLTF manipulation in both cell lines, the cells were washed and dissolved in TRIzol reagent for preservation. Transcriptome sequencing was performed by SeqHealth Technology Co. Ltd, the results are shown in Table S8.

### 2.11 Chromatin immunoprecipitation (ChIP) assay

A kit from Active Motif (53008) was used for ChIP assays in accordance with the manufacturer's instructions. In short, the formaldehyde‐immobilised cells were able to cross‐link and protect the interaction between proteins and DNA. The DNA is then cut into small fragments, which can be cut into relatively uniform size fragments by ultrasound or enzyme digestion, and the protein of interest and DNA interaction complex are immunized by antibodies. Following the decrosslinking, Proteinase K is used to remove the protein and recover the target DNA fragment. Analysis of the recovered DNA reveals which DNA sequence the protein binds to.

### 2.12 RNA pull‐down assay and RNA immunoprecipitation (RIP) assay

Full‐length or truncated LINC01088 specific probes and negative control (NC) probe were synthesized from GeneCreate Biological Engineering. RNA pull‐down assay was conducted by using a Pierce Magnetic RNA‐Protein Pull‐Down Kit (Thermo Fisher Scientific) according to the manufacturer's instructions. The co‐precipitated proteins eluted from the beads were separated by SDS‐PAGE and then silver stained by using a Fast Silver Stain Kit (P0017S, Beyotime) following the manufacturer's recommendations.

For RIP assay, the Magna RIP RNA‐Binding Protein Immunoprecipitation Kit (Millipore) was used according to the manufacturer's instructions. Cell lysates obtained from 3 to 4 × 10^7^ cells were subjected to immunoprecipitation at 4°C overnight with primary antibodies against HLTF (14286‐1‐AP, Proteintech). The isotype control was a homologous IgG. TRIzol reagent was used for extracting RNA samples, which were then tested through RT‐qPCR.

### 2.13 Immunoprecipitation (IP) assay

IP was carried out using indicated antibodies and IgG by the manufacturer's instruction. In brief, cell lysates were added with Protein A + G Agarose (Beyotime Biotechnology) and incubated with appropriate antibody. The immunocomplexes were examined by Western blot according to the protocol above.

### 2.14 Malondialdehyde (MDA), iron, lipid ROS, and glutathione (GSH)

The Lipid Peroxidation MDA Assay Kit (S0131 M, Beyotime) was used to determine the relative MDA concentration in cell lysate according to the manufacturer's instructions. In short, glioma cells in 10‐cm dishes with the treatment of erastin or DMSO for 48 h were lysed via western blotting and immunoprecipitation (IP) (P0013, Beyotime). Then, cell homogenates were centrifugated at 13 000 g for 10 min, and 100 µL of obtained supernatant was mixed with 200 µL of MDA working solution and incubated at 100°C for 15 min. After cooling to room temperature, the absorbance of each mixture was measured at 532 nm. Intracellular ferrous iron (Fe^2+^) level was measured by using the iron assay kit (ab83366, Abcam) according to the manufacturer's instructions. Briefly, glioma cells were seeded in a 10‐cm plate and treated with erastin or DMSO for 48 h. Then, cells were harvested, washed in ice cold PBS, and homogenized in 5× volumes of iron assay buffer on ice. The supernatant was obtained after centrifugation (13 000 × g, 10 min) at 4°C, mixed with iron reducer, and incubated at room temperature for 30 min. Subsequently, each sample mixed with 100 µL of iron probe was incubated at room temperature in the dark for 1 h. The absorbance at 593 nm was measured immediately using a colorimetric microplate reader. Lipid ROS level was detected by flow cytometry via BODIPY‐C11 dye (D3861, Thermo Fisher Scientific). Glioma cells were seeded in 6‐well plates and treated with erastin or DMSO for 48 h. After washing with PBS, cells were stained with 2 mL complete medium containing 5 µM of BODIPY‐C11 dye and incubated at 37°C for 30 min in the dark. Then, cells were washed thrice with PBS to remove excess labelling mixture followed by resuspending in 200 µL medium. Oxidation of BODIPY‐C11 resulted in a shift of the fluorescence emission peak from 590 to 510 nm proportional to lipid ROS generation. GSH and GSSG Assay Kit (S0053, Beyotime) was used to determine the relative GSH/GSSG concentration in cell lysate according to the manufacturer's instructions. GSSG is reduced to GSH by glutathione reductase, and GSH can react with colour producing substrate DTNB to produce yellow TNB and GSSG. When the reaction system is properly formulated and the two reactions are combined, the total glutathione (GSSG + GSH) is equivalent to a rate‐limiting factor for colour production, and the amount of total glutathione determines the amount of yellow TNB formation. The amount of total glutathione can be calculated by measuring A412. The content of GSSG can be determined by removing GSH from the sample with appropriate reagents, and then using the reaction principle mentioned above. The amount of GSSG can be calculated by subtracting the amount of GSSG from the amount of total glutathione (GSSG + GSH).

### 2.15 Animal experiments

All animal experiments were approved by the Committee on Ethics of Animal Experiments of Huazhong University of Science and Technology and were performed according to the NIH animal care guidelines. Six‐week‐old female BALB/c‐nude mice aged 6 weeks (BIONT) were used to construct intracranial xenograft models. Briefly, 5 ×10^5^ U87MG cells stably expressing firefly luciferase (Fluc) with the indicated treatments were injected into the mouse brain at 2 mm lateral, 2 mm posterior to the bregma, and 2 mm depth via a stereotaxic apparatus. Tumour growth was monitored by bioluminescence imaging (Bruker Corporation). The weight and survival time of the mice were carefully recorded. Tumour tissues harvested from the mice were used for the corresponding staining. Subcutaneous xenografts were suspended in 100 µL serum‐free medium and subcutaneously injected into BALB/c nude female mice (six mice per group). We measured the tumour volume, body weight, and the maximum and weight of the tumours. Protein and nucleic acids were extracted from the tumour tissue for corresponding detection.

### 2.16 Bioinformatics analysis

The transcript data of glioma samples together with clinical information was downloaded from The Cancer Genome database (TCGA, https://xenabrowser.net/heatmap/), the Chinese Glioma Genome Atlasm RNAseq_693 database (http://www.cgga.org.cn/index.jsp), and GEO (GSE4290, GSE7696, GSE15824 and GSE50161). The transcript data of normal brain tissues was downloaded from the Genotype‐Tissue Expression (GTEx) database (https://gtexportal.org/home/). Ferroptosis‐related genes were obtained from FerrDbV2 (http://www.zhounan.org/ferrdb/current/). Promoter binding sites were predicted using JASPAR and hTFtarget databases.

### 2.17 Statistical Analysis

Graphpad prism 8.0 was applied to perform statistical analyses. The data were presented as mean ± standard deviation. Spearman rank correlation was used for correlation analysis. Statistical comparisons between two groups were carried out by Student's *t*‐test. Mouse survival data was analysed by the Kaplan–Meier log‐rank test. *p *< 0.05 was considered to reflect a statistically significant difference. All experiments were repeated at least three times.

## 3 RESULTS

### 3.1 Ferroptosis related lncRNA LINC01088 is significantly overexpressed in GBM

**FIGURE 1:**
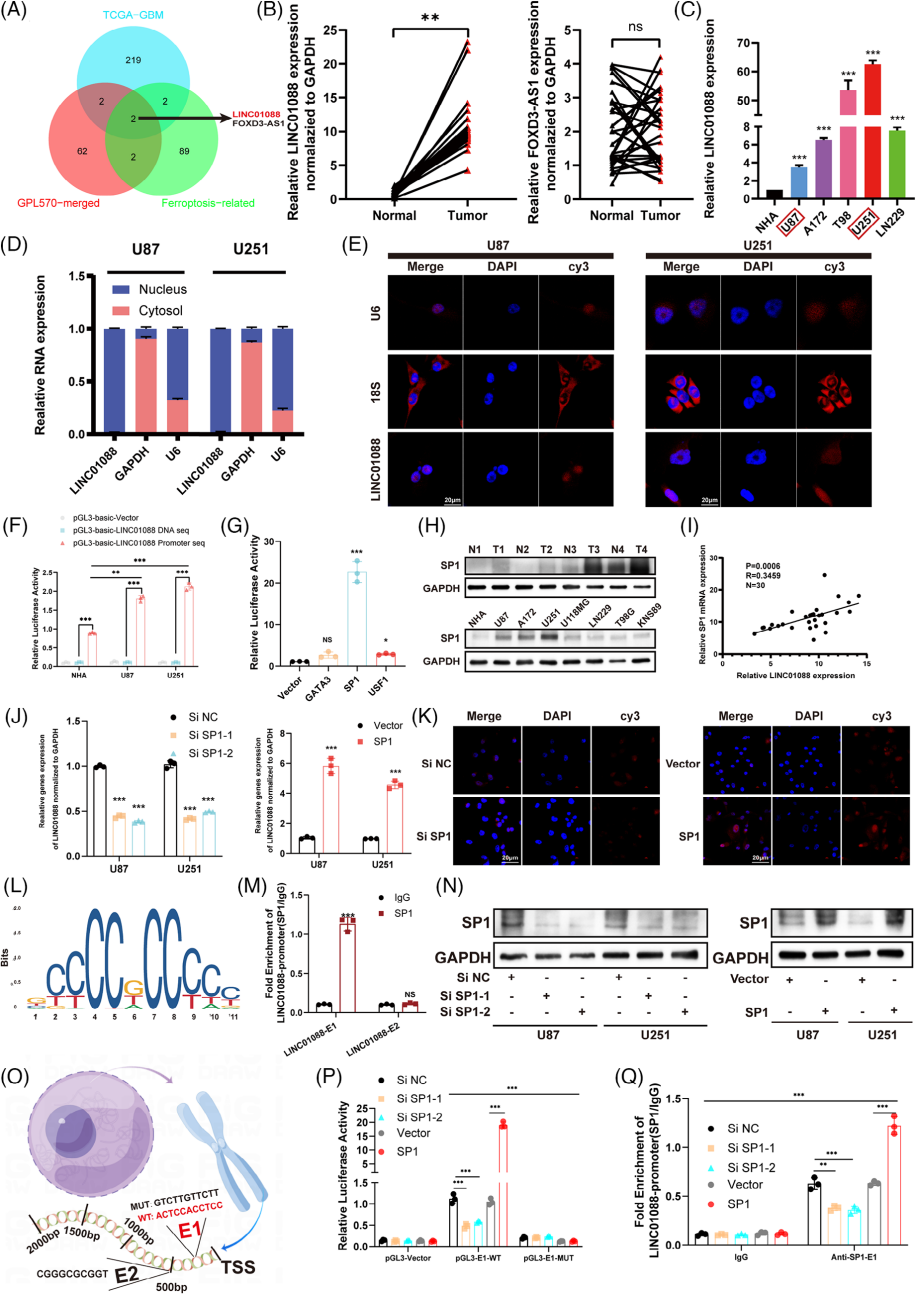
SP1 promote the expression of ferroptosis related lncRNA LINC01088 in glioblastoma multiforme (GBM). (A) Venn diagram is used to represent the intersection LncRNAs of three data sets: TCGA‐GBM, GPL570‐merged and ferroptosis‐related. (B) The relative expression of LINC01088 and FOXD3‐AS1in GBM tissue and normal tissue samples. (C) The relative expression of LINC01088 in normal astrocytes and common GBM cell lines. (D) The relative expression of LINC01088 in the nucleus and cytoplasm of U87MG and U251 cell lines. (E) FISH clearly demonstrated the nucleus subcellular localization of LINC01088 in U87MG and U251 cell lines. (F) The control plasmid with LINC01088 itself sequence and full promoter sequence was transfected into NHA, U87MG and U251 cells to determine the relative fluorescence intensity. (G) The plasmid carrying the LINC01088 promoter region was co‐transfected into U87MG cells with three transcription factor plasmids, respectively, and the relative fluorescence intensity was measured. (H) SP1 protein expression levels in tissues, NHA and GBM cell lines. (I) LINC01088 was positively correlated with the relative expression of SP1 in GBM tissues. (J) The relative expression of LINC01088 was detected after transfection with Si‐SP1 and SP1 plasmid in vitro. (K) FISH showed the relative intensity of LINC01088 after transfection with Si‐SP1 and SP1 plasmid in vitro. (L) SP1‐bound Motif. (M) The concentration of SP1 in the binding region of two predicted LINC01088 promoters was detected by ChIP‐PCR. (N) The protein level of SP1 was detected after transfection of Si‐SP1 and SP1 plasmid in vitro. (O) The JASPAR database predicts two promoter binding region schemata. (P) The plasmids carrying the LINC01088 promoter region E1‐MUT (mutant) and WT (wild type) were co‐transfected into U87MG cells with control plasmids, Si‐SP1 and SP1 plasmids, respectively, and the relative fluorescence intensity was measured. (Q) The relative abundance level of SP1 antibody in the promoter binding region of LINC01088‐E1 was detected by ChIP‐PCR after transfection of Si‐SP1 or SP1 plasmid in vitro. *p < 0.05, **p < 0.01, ***p < 0.001, ns: not significant.

In this study, we conducted a comprehensive analysis of publicly available datasets, including TCGA‐GBM, FerrDb, and four GBM datasets (GSE4290, GSE7696, GSE15824 and GSE50161) from the GEO database, using the GPL570 platform. Using R software, we focussed on identifying the upregulated lncRNAs as potential therapeutic targets or prospective biomarkers. Furthermore, we conducted an extensive literature review to compile a comprehensive list of the lncRNAs associated with ferroptosis. The candidate lncRNAs are shown in Tabel S9. Employing a Venn diagram tool, we identified two overlapping lncRNAs, LINC01088 and FOXD3‐AS1, which were common between the differentially expressed lncRNAs and ferroptosis‐related lncRNAs (Figure 1A). To further validate the significance of these target molecules, we assessed their expression levels in 30 clinical GBM and 30 normal brain tissue samples. Intriguingly, the analysis revealed a significant upregulation of LINC01088 in GBM tissues compared to that in the control group, whereas the expression of FOXD3‐AS1 did not exhibit a significant difference (Figure 1B). Therefore, LINC01088 was selected to be the primary focus of subsequent investigation. The relative expression levels of LINC01088 were examined in both normal human astrocytes (NHA) and commonly used GBM cell lines (**Figure** **1C**). On the basis of the specific expression levels of LINC01088, the U87MG (with the lowest relative expression of LINC01088) and U251 (with the highest relative expression of LINC01088) cell lines were selected as the primary cell models for subsequent investigations. Furthermore, to gain a deeper understanding of the potential mechanisms underlying LINC01088 activity, it is essential to determine its subcellular localization in GBM cells. Cell fractionation experiments were conducted to separate the nuclear and cytoplasmic compartments. The results indicated that LINC01088 was predominantly localized in the nucleus (Figure 1D). Moreover, a FISH analysis provided compelling evidence to support this conclusion (Figure 1E).

### 3.2 SP1 promotes the expression of LINC01088 in GBM

To elucidate the mechanisms underlying the upregulation of LINC01088 inGBM, we employed dual‐luciferase reporter constructs containing the full‐length sequence and promoter sequence of LINC01088. The dual‐luciferase reporter assay demonstrated that the reporter constructs containing the LINC01088 promoter sequence exhibited a marked increase in relative fluorescence intensity compared to those containing the full‐length sequence alone (Figure 1F), which suggests that the expression of LINC01088 may be regulated at the transcriptional level. Several studies have shown that transcription factors play important roles in the regulation of lncRNA expression. Therefore, we used the JASPAR (http://jaspar.genereg.net/) and the hTFtarget databases (http://bioinfo.life.hust.edu.cn/hTFtarget) to perform bioinformatics analysis of the promoter region of LINC01088 to predict potential binding sites for transcription factors. Dual‐luciferase reporter assays were then used to detect the binding activity of the top three predicted transcription factors. GBM cell lines were transfected with a luciferase plasmid containing the promoter region of LINC01088 and plasmids containing either individual transcription factors or the control sequence. SP1 showed the maximum luciferase activity (Figure 1G). An analysis of the TCGA‐GBM dataset demonstrated a significant upregulation of SP1 in GBM cells (Figure S1). This observation was further validated in GBM cell lines and tissue samples (Figure 1H and Figure S2). Moreover, in 30 GBM tissue sample, a positive correlation was identified between SP1 and LINC01088 expression (Figure 1I). SP1 knockdown resulted in reduced LINC01088 expression, whereas SP1 increased its levels (Figure 1J). The concordance between these findings was visually confirmed by FISH analysis (Figure 1K). Furthermore, a bioinformatics analysis using the JASPAR database predicted potential binding sites (E1 and E2) and motifs for the interaction between SP1 and the promoter regions of LINC01088 (Figure L and O). Chromatin immunoprecipitation (ChIP) experiments revealed significant enrichment of SP1 antibody at the E1 site (Figure 1M). In vitro transfection of two Si‐SP1 constructs, and one overexpression plasmid led to the selection of Si‐SP1‐1 for subsequent SP1 knockdown experiments based on western blotting results (Figure 1N). Further confirmation was provided by dual luciferase reporter assays (Figure 1P) and ChIP experiments (Figure 1Q), which demonstrated that SP1 specifically regulates the expression of LINC01088 by binding to the E1 region of its promoter.

### 3.3 LINC01088 promotes malignant proliferation and inhibits ferroptosis in GBM cells

**FIGURE 2:**
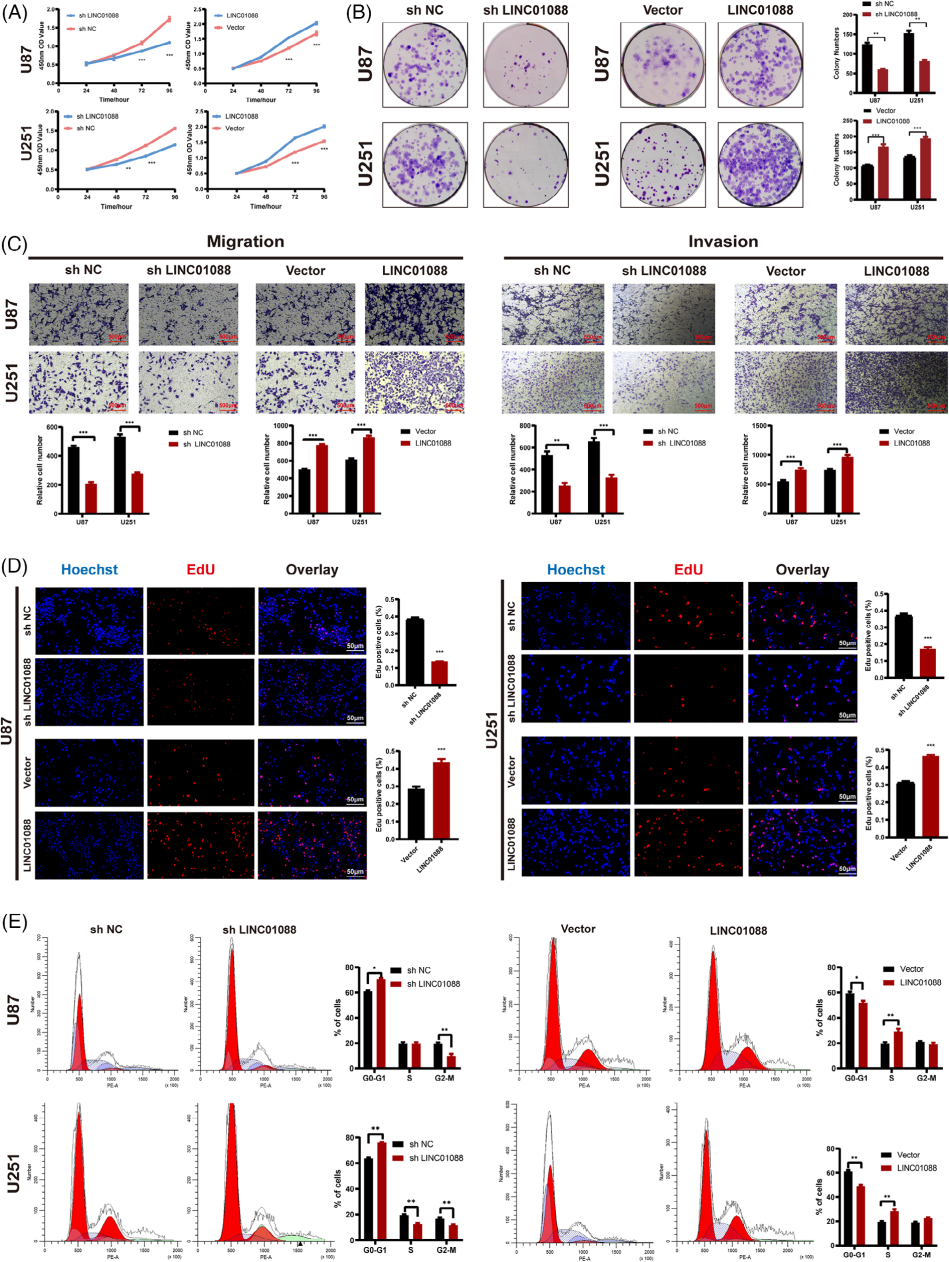
LINC01088 inhibits the proliferation, invasion, and migration of glioblastoma multiforme (GBM) in vitro. (A) The growth curves of transfected U87MG and U251 cells were determined by CCK8 assays. (B) The colony formation assays were performed in transfected U87MG and U251 cells. (C) The transwell assays showed the migration and invasion abilities of transfected U87MG and U251 cells. (D) The proliferation of transfected U87MG and U251 cells was detected by EdU staining assays. (E) Cell cycle distributions of transfected U87MG and U251 cells were measured by flow cytometry. *p < 0.05, **p < 0.01, ***p < 0.001, ns: not significant.

**FIGURE 3:**
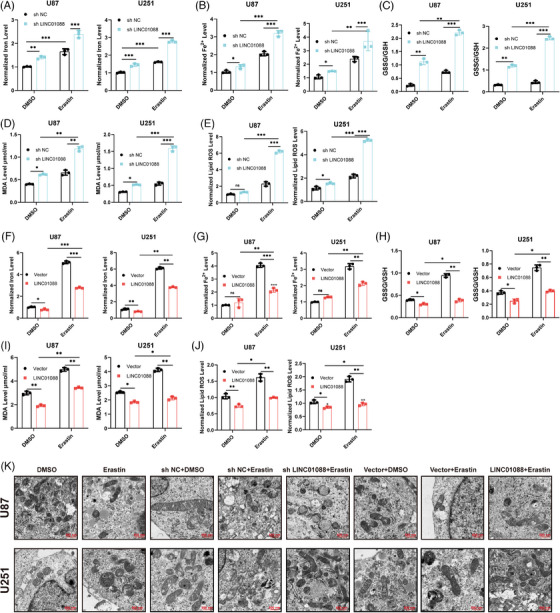
LINC01088 inhibit ferroptosis in vitro. (A–E) U87MG and U251 cells stably knock downing LINC01088 were treated with erastin (10µM) for 48 h, total iron (Fe), ferrous iron (Fe^2+^) was measured by iron detection assays (A and B), GSH/GSSG determined by GSH assays (C), intracellular MDA was determined by MDA assays (D), lipid ROS accumulation was operated by C11‐BODIPY staining (E). (F–J) U87MG and U251 cells stably overexpression LINC01088 were treated with erastin (10 µM) for 48 h, total iron (Fe), ferrous iron (Fe^2+^) was measured by iron detection assays (F and G), GSH/GSSG determined by GSH assays (H), intracellular MDA was determined by MDA assays (I), lipid ROS accumulation was operated by C11‐BODIPY staining (J). (K) Transmission electron microscopy was performed to evaluate the ultrastructural changes of mitochondria in U87MG and U251 after treated with erastin (10µM) for 48 h. *p < 0.05, **p < 0.01, ***p < 0.001, ****p < 0.0001, ns: not significant.

Gain‐ and loss‐of‐function experiments were performed to elucidate the role of LINC01088 in the tumorigenesis and progression of GBM. We stably knocked down and overexpressed LINC01088 in U87MG and U251 cell lines, selecting the sh2 construct with the most efficient knockdown for subsequent experiments. The effect of LINC01088 on GBM cell proliferation was evaluated using CCK‐8, colony formation, EdU and cell cycle flow cytometry assays. The results demonstrated that LINC01088 knockdown (LINC01088‐SH) inhibited GBM cell proliferation, while LINC01088 overexpression (LINC01088‐OE) promoted GBM cell proliferation. Transwell assays were conducted to assess the effect of LINC01088 on GBM cell invasion and migration. The results indicated that LINC01088‐OE enhanced GBM cell invasion and migration, whereas LINC01088‐SH weakened GBM cell invasion and migration (Figure 2A–E). Taken together, these findings suggested that LINC01088 promotes GBM cell proliferation, invasion, and migration. The selected lncRNAs were associated with ferroptosis. A Gene Set Enrichment Analysis (GSEA) analysis of LINC01088 further revealed that differentially expressed genes were mainly enriched in the oxidative stress pathway (Figure S3), which is closely related to apoptosis. Therefore, we hypothesized that LINC01088 may be involved in GBM cell apoptosis. The results demonstrated that knockdown of LINC01088 led to a significant increase in the proportion of GBM cells damaged by erastin‐induced toxicity. We found that LINC01088‐SH increased the intracellular concentrations of total iron (Fe), ferrous iron (Fe^2+^), lipid ROS, glutathione disulfide (GSSG) to GSH ratio, and MDA in GBM cells after treatment with erastin (Figure 3A–E). Transmission electron microscopy (TEM) analysis revealed severe mitochondrial shrinkage in GBM cells treated with LINC01088‐SH (Figure 3K). Overall, these results indicate that LINC01088‐SH enhanced the sensitivity of GBM cells to apoptosis. As expected, LINC01088‐OE led to decreases in the intracellular concentrations of total iron (Fe), ferrous iron (Fe^2+^), lipid ROS, the GSSG to GSH ratio, and MDA in GBM cells after treatment with erastin (Figure 3F–J). A TEM analysis revealed that the altered mitochondrial ultrastructure in GBM cells was recovered after LINC01088‐OE treatment (Figure 3K). In addition, the rescue experiments found that the trend of LINC01088‐OE enhancing the GBM malignant progression can be offset by erastin. These results indicate that LINC01088‐OE promoted GBM malignant proliferation though resistance to ferroptosis.

### 3.4 HLTF is a key LINC01088‐interacting protein

**FIGURE 4:**
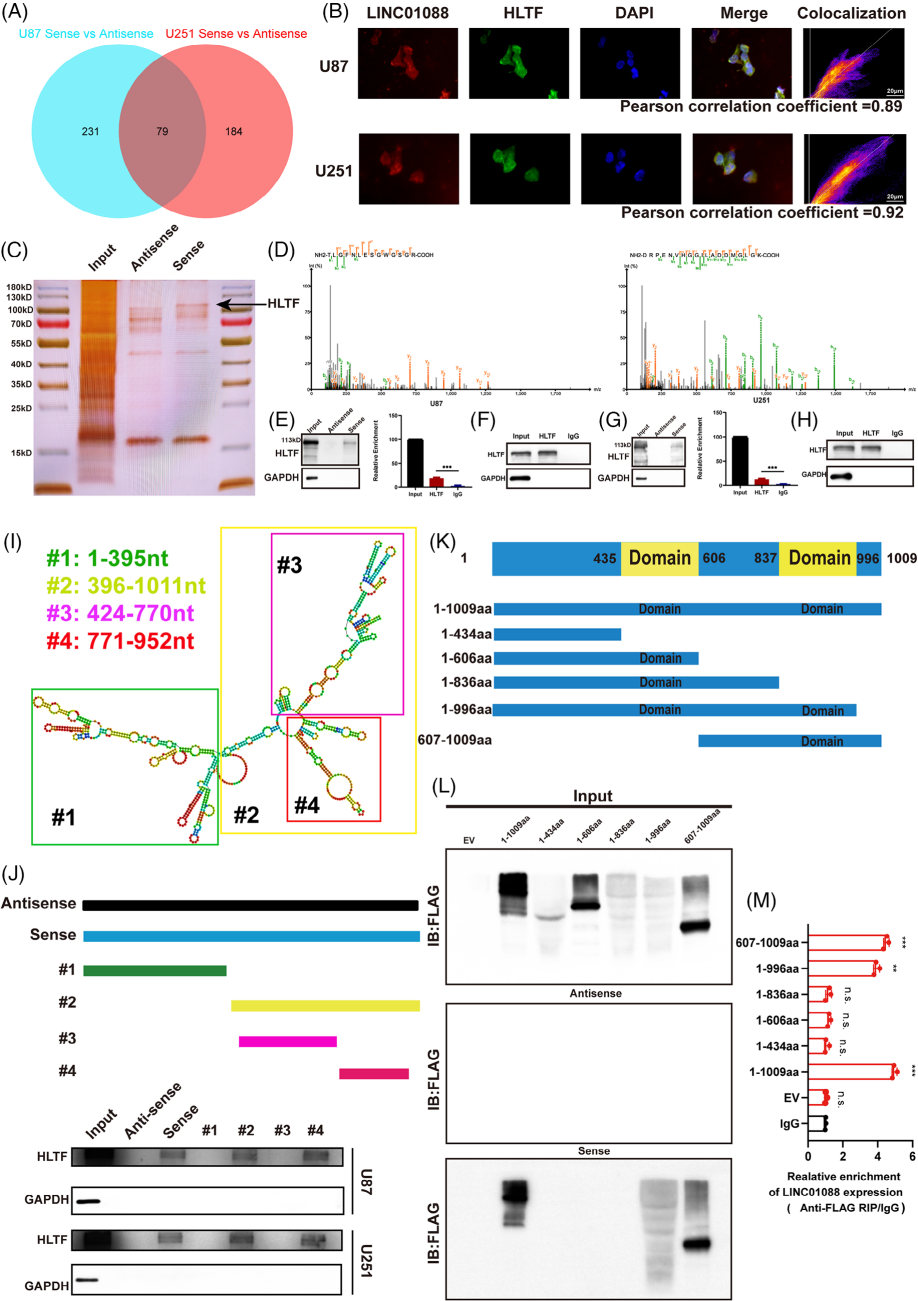
LINC01088 binds to HLTF protein. (A) Venn diagram was used to analyse the differentially binding proteins of U87MG and U251 cell lines by magnetic bead mass spectrometry after RNA pulldown experiment. (B) Co‐localization of LINC01088 and HLTF proteins in U87MG cells and U251 cells. (C) After RNA pulldown experiment, protein samples were taken for silver staining test, Sense: LINC01088 biotin probe, Antisense: LINC01088 antisense chain biotin probe. (D) Secondary profile of HLTF in U87MG and U251 cell lines in mass spectrometry. (E and G) The interaction between HLTF and LINC01088 was verified by Western blot analysis of protein samples obtained from RNA pulldown in U87MG cells (E) and U251 cells (G). (F and H) RIP detection was performed in U87MG cells (F) and U251 cells (H) using IgG or anti‐HLTF antibody. Western blot analysis confirmed the interaction between HLTF and LINC01088, qRT‐PCR was performed to detect the abundance of LINC01088. (I) The predicted secondary structure of LINC01088. (J) Schematic diagram of 4 truncated sequences of LINC01088 and the results of RNA pulldown experiment. (K) The diagrams of Flag‐tagged full‐length or truncation plasmids with various assembled domains of HLTF protein. (L) Western blot analysis verified the interaction between each truncated HLTF protein and LINC01088 (M) The enrichment of LINC01088 was detected by RIP and qRT‐PCR. *p < 0.05, **p < 0.01, ***p < 0.001, ****p < 0.0001, ns: not significant.

Given the subcellular localization of LINC01088, which is mainly within the cell nucleus, it is likely to function through interactions with RNA‐interacting proteins that regulate gene expression through various mechanisms. To explore this, we conducted RNA pull‐down assays to identify potential protein partners of LINC01088. The antisense strand of LINC01088 served as the negative control. A mass spectrometry analysis revealed that LINC01088 interacted with numerous proteins in U87MG and U251 cells (Figure 4A). Colocalization experiments also demonstrated that LINC01088 and HLTF colocalized prominently within the nuclei of U87MG and U251 cells (Figure 4B). The silver staining results revealed a distinct protein band around 110–115 kDa, which was identified as HLTF by mass spectrometry (Figure 4C). The secondary structure of the protein is shown in Figure 4D. Furthermore, RNA pull‐down assay using biotin‐labelled LINC01088 with antisense LINC01088 as a negative control confirmed the interaction between LINC01088 and HLTF at the endogenous level in U87MG and U251 cells (Figure 4E–G). RNA immunoprecipitation (RIP) assays using an anti‐HLTF antibody further validated the interaction between HLTF and LINC01088 in U87MG and U251 cells (Figure 4F–H). Thus, HLTF was confirmed as a novel binding partner of LINC01088. To identify the specific regions within LINC01088 that contribute to HLTF binding, we predicted the secondary structure of LINC01088 using an RNA‐fold web server (http://rna.tbi.univie.ac.at/cgi‐bin/RNAfold.cgi) (Figure 4I). We constructed four different deletion fragments of LINC01088 and found that one HLTF‐specific binding sequence within LINC01088 (771‐952 nt) was essential for its efficient binding to HLTF in GBM cells (Figure 4J). Similarly, we examined the specific regions within HLTF that are responsible for binding to LINC01088 by constructing five different deletion fragments of HLTF (Figure 4K). The second domian (837‐996 aa) of HLTF exhibited the same binding efficiency LINC01088 as the full‐length HLTF (Figure 4L). Through RIP assays, we confirmed that LINC01088 binds to the domain (837–996 aa) of HLTF (Figure 4 M). These results indicate that the region within the HLTF from aa 837 to 996 interacts with the region of LINC01088 from 771 to 952 nt.

### 3.5 LINC01088 stabilizes HLTF by facilitating USP7‐mediated protection from ubiquitin degradation

**FIGURE 5:**
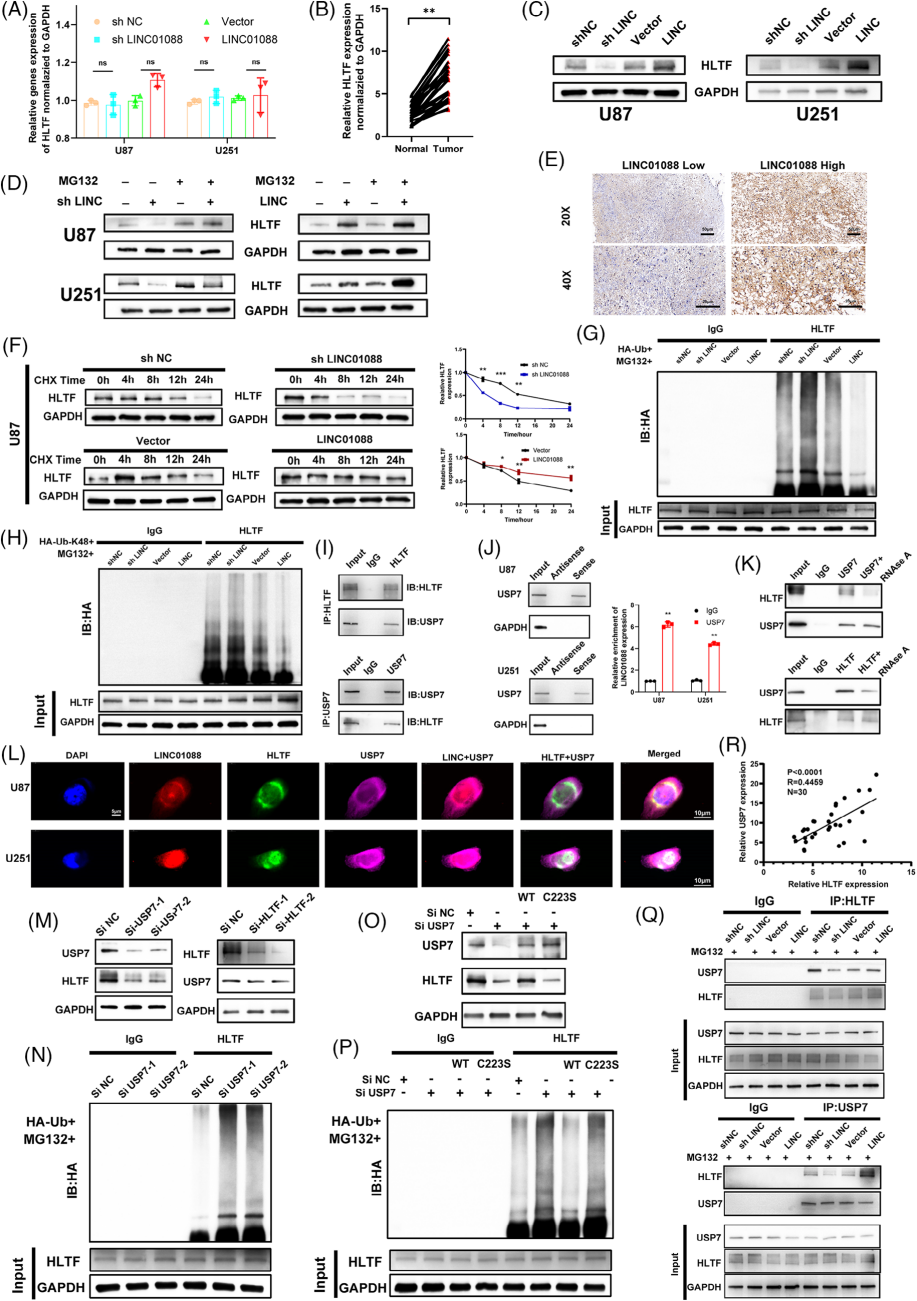
LINC01088 acts as a USP7/HLTF interaction support platform to protect HLTF from ubiquitin/proteasome degradation. (A) qRT‐PCR was used to detect HLTF mRNA levels in GBM cell lines after knockdown and overexpression of LINC01088. (B) qRT‐PCR was used to detect the relative expression of HLTF in tissues. (C) WB was used to detect HLTF protein levels in GBM cell lines after knockdown and overexpression of LINC01088. (D) After MG132 (20 µM) treatment for 12 h, the expression level of HLTF protein in U87MG and U251 cells was detected by WB. (E) Immunohistochemistry showed HLTF protein expression levels in tissue samples with high or low expression of LINC01088. (F) The protein expression level of HLTF was detected by WB after 60 µg/mL CHX treatment in U87MG cells. (G–H) IP experiments showed that down‐regulation of LINC01088 increased the ubiquitination level of HLTF (G) and K48 (H), while overexpression of LINC01088 had the opposite effect. (I) WB was used to analyse the Co‐IP detection with IgG antibody or HLTF antibody or USP7 antibody. (J) The specific association between USP7 and LINC01088 was verified by RNApulldown and RIP. (K) Co‐IP analysis showed that LINC01088 may be involved in the binding of HLTF and USP7. (L) The co‐localization of LINC01088, HLTF and USP7 proteins in U87MG and U251 cells was detected by immunofluorescence and FISH. (M) knockdown of USP7 reduced HLTF protein levels, while downregulation of HLTF had no effect on USP7 protein levels. (N) IP detection showed that knocking down USP7 could enhance the ubiquitination level of HLTF. (O–P) Western blot analysis confirmed USP7 and HLTF protein levels (O) and HLTF ubiquitination levels (P) in the si‐NC and si‐USP7 groups rescued by wild‐type or inactive mutant USP7. (Q) Low LINC01088 decreased HLTF‐USP7 interaction, while overexpression of LINC01088 enhanced HLTF‐USP7 interaction. (R) mRNA expression correlation analysis of USP7 and HLTF in tissue. *p < 0.05, **p < 0.01, ***p < 0.001, ****p < 0.0001, ns: not significant.

To elucidate the consequences of the LINC01088‐HLTF interaction, we investigated the impact of LINC01088‐SH and LINC01088‐OE on HLTF mRNA and protein levels (Figure 5A,C). LINC01088 did not influence the mRNA level of HLTF in GBM cell lines, but LINC01088‐SH led to a significant decrease in HLTF protein levels, whereas LINC01088‐OE resulted in an increase in HLTF protein levels. Additionally, we examined HLTF expression in GBM tissues and adjacent non‐tumour tissues using qRT‐PCR and immunohistochemistry. The results revealed that HLTF mRNA levels were higher in GBM tissues than in non‐tumour tissues, and tissues with elevated LINC01088 levels exhibited upregulated HLTF protein expression (Figure 5B,E). A western blotting analysis also revealed that HLTF protein expression was upregulated in GBM cell lines (Figure S4). To explore whether LINC01088 affects HLTF protein levels by regulating its degradation, we assessed the turnover rate of HLTF in GBM cells expressing LINC01088‐SH or LINC01088‐OE using the protein synthesis inhibitor cycloheximide (CHX). Our findings demonstrated that LINC01088‐SH accelerated the turnover rate of HLTF, whereas LINC01088‐OE reduced the turnover rate of HLTF in the GBM cell lines (Figures 5F and S5). Moreover, the effect of LINC01088 on HLTF protein levels was reversed by treatment with the proteasome inhibitor, MG132 (Figure 5D). Furthermore, ubiquitination experiments further revealed that downregulation of LINC01088 increased the overall ubiquitination and K48‐linked ubiquitination levels of the HLTF protein. Conversely, upregulation of LINC01088 reduced the overall ubiquitination and K48‐linked ubiquitination levels of HLTF (Figure 5G,H). These observations suggest that LINC01088 may stabilize HLTF at the protein level by inhibiting proteasome‐mediated ubiquitination and degradation. The process of ubiquitination is tightly regulated, with deubiquitinases playing a crucial role in maintaining the balance among ubiquitination modifications. To further investigate the DUBs involved in HLTF ubiquitination, we performed a mass spectrometry analysis after RNA pull‐down. Interestingly, among all participants, only one deubiquitinase, ubiquitin‐specific protease (USP)‐7, was identified. Previous studies have also indicated that USP7 acts as a deubiquitinase of HLTF, regulating its ubiquitination levels.^30^ The specific interaction between HLTF and USP7 was confirmed by co‐immunoprecipitation and western blotting (Figure 5I). Furthermore, RNA pull‐down and RIP assays further revealed an interaction between USP7 and LINC01088 (Figure 5J). Through immunofluorescence and FISH experiments, we observed the colocalization of LINC01088, HLTF, and USP7 in the nuclei of U87 and U251 cells (Figure 5L). Additionally, when analyzing TCGA‐GBM data (Figure S6) and clinical GBM tissue samples (Figure 5R), we found a significant positive correlation between the relative expression levels of USP7 and HLTF mRNA. Notably, knockdown of USP7 significantly decreased HLTF protein levels and increased HLTF ubiquitination, whereas HLTF did not affect the expression of USP7 protein (Figure 5 M and N). To determine whether the effect of USP7 on HLTF depends on its deubiquitinase activity, we assessed the impact of wild‐type and the catalytically inactive C223S mutant USP7 on HLTF. As shown in Figure 5O ,P, wild‐type USP7, but not the inactive mutant, rescued the decrease in HLTF protein levels and increase in ubiquitination caused by USP7 knockdown, demonstrating that USP7 is a specific deubiquitinase for HLTF. Recent studies have reported that lncRNAs can serve as scaffolds for protein‐protein interactions.^31^ Therefore, we investigated whether LINC01088 is required for the interaction between HLTF and USP7. The results indicated that the interaction between HLTF and USP7 was disrupted by ribonuclease A treatment (Figure 5K) and LINC01088 knockdown (Figure 5Q). Conversely, LINC01088 overexpression enhanced the interaction between HLTF and USP7 (Figure 5Q). Overall, these results suggest that LINC01088 functions as a scaffold platform for the HLTF/USP7 interaction, protecting HLTF from degradation by the ubiquitin/proteasome system.

### 3.6 HLTF Promotes SLC7A11 transcription in GBM cell

**FIGURE 6:**
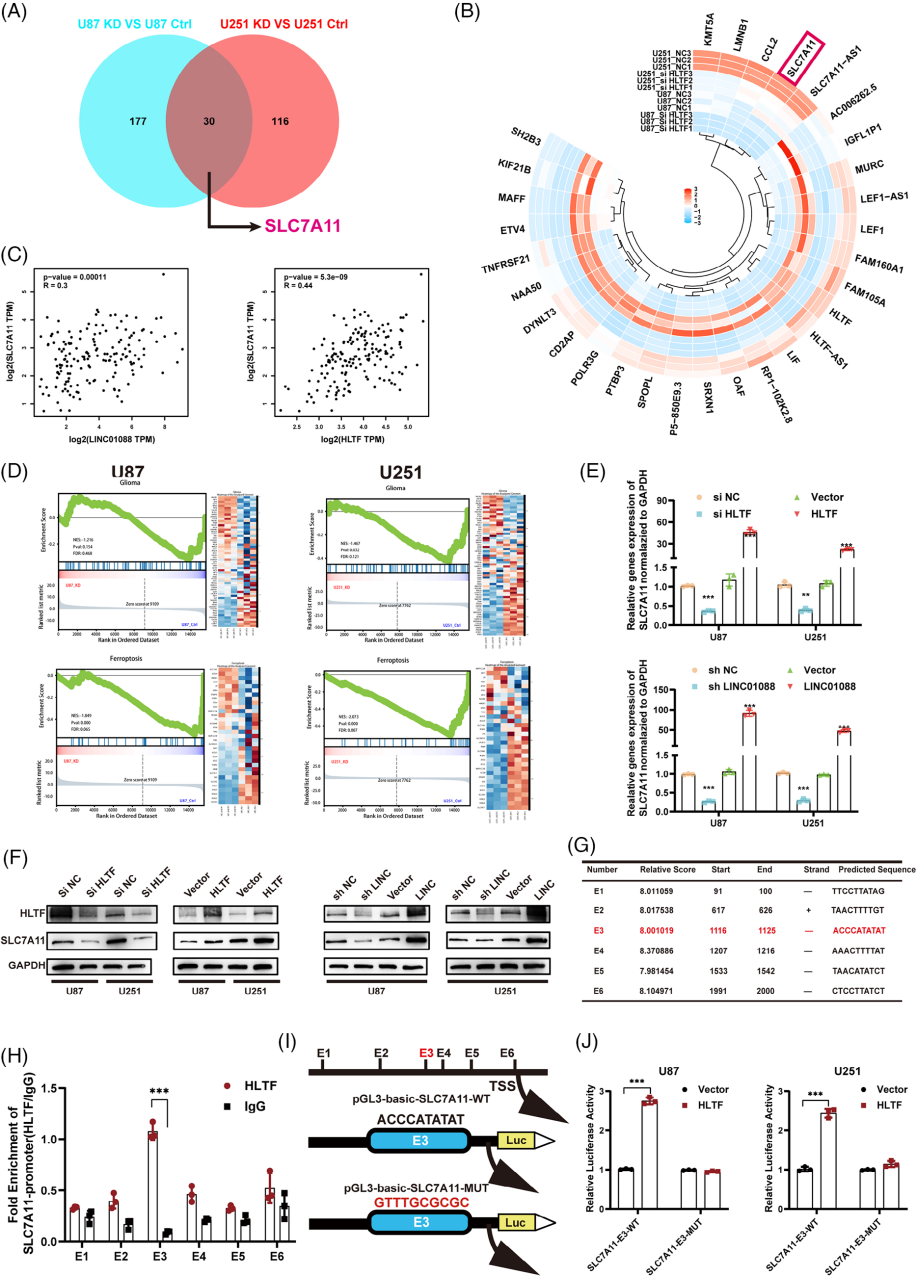
HLTF up‐regulates SLC7A11 transcription. (A) After si‐HLTF, differentially down‐regulated gene intersection in the U87MG and U251 cell lines. B: Heat map showing the expression of 30 differential genes in each sample. (C) GEPIA database analysis of correlation between SLC7A11 and LINC01088 or HLTF. (D) GSEA analysis showed significant enrichment of ferroptosis and glioma pathways in U87 and U251 cell lines. (E) The relative expression of SLC7A11 was detected by qRT‐PCR after knockdown and overexpression of LINC01088 and HLTF in U87MG and U251 cell lines. (F) The protein expression of SLC7A11 was detected by WB after knockdown and overexpression of LINC01088 and HLTF in U87MG and U251 cell lines. (G) JASPAR database predicted 6 possible transcription factor binding sites. (H) ChIP‐qPCR was used to analyse the enrichment degree of HLTF antibody in 6 possible transcription factor binding sites. (I) Schematic diagram of transcription factor regulation and display of wild type and mutant sequences in region E3. (J) The wild type plasmid or mutant plasmid was co‐transfected with HLTF plasmid, and the relative fluorescence intensity was subsequently measured in U87MG and U251cell lines. *p < 0.05, **p < 0.01, ***p < 0.001, ****p < 0.0001, ns: not significant.

To further explore the underlying molecular mechanisms, we conducted transcriptome sequencing in U87MG and U251 cells following HLTF knockdown, with a particular focus on the downregulated differentially expressed genes. The intersection of downregulated genes in both cell lines revealed 30 common genes (Figure 6A), which are depicted in a heatmap showing their relative expression levels in differentially treated samples of the U87 and U251 cells (Figure 6B). Among these, SLC7A11 has emerged as a pivotal gene in ferroptosis. Analyses using the GEPIA database demonstrated a positive correlation between SLC7A11, LINC01088 and HLTF (Figure 6C). Furthermore, a GSEA enrichment analysis of the downregulated differentially expressed genes from the sequencing data revealed the significant enrichment of the ferroptosis pathway in both the U87MG and U251 cells (Figure 6D). Therefore, we hypothesized that SLC7A11 is a target gene regulated by HLTF. To validate whether SLC7A11 is indeed under the control of HLTF, qRT‐PCR was used to examine the impact of LINC01088 and HLTF on SLC7A11 expression in U87 and U251 cells. The results demonstrated that knockdown of LINC01088 and HLTF led to a decrease in SLC7A11 expression, whereas overexpression of LINC01088 and HLTF had the opposite effect (Figure 6E). Moreover, Western blotting analysis showed that downregulation of LINC01088 and HLTF resulted in decreased SLC7A11 protein levels, whereas overexpression of LINC01088 and HLTF led to increased SLC7A11 protein levels (Figure 6F). Furthermore, transcription factors (TFs) can specifically bind to certain regions within the promoters of downstream target genes and, with the assistance of other cofactors, regulate their transcription. Using bioinformatic analyses, six potential TF‐binding sites (TFBS) were predicted (Figure 6G). ChIP experiments confirmed the significant enrichment of the HLTF antibody at site E3 (Figure 6H). A schematic representation of TF regulation and display of the wild‐type and mutant sequences of the E3 region are also provided (Figure 6I). Dual‐luciferase reporter assays demonstrated that, in both U87MG and U251 cell lines, the wild‐type E3 sequence exhibited significantly higher fluorescence intensity than the mutant E3 sequence (Figure 6J). Collectively, these collective findings support the conclusion that HLTF modulates SLC7A11 transcription by binding to the E3 region of the SLC7A11 promoter.

### 3.7 LINC01088 interacts with HLTF to upregulate SLC7A11 transcription and inhibit ferroptosis in GBM cell

**FIGURE 7:**
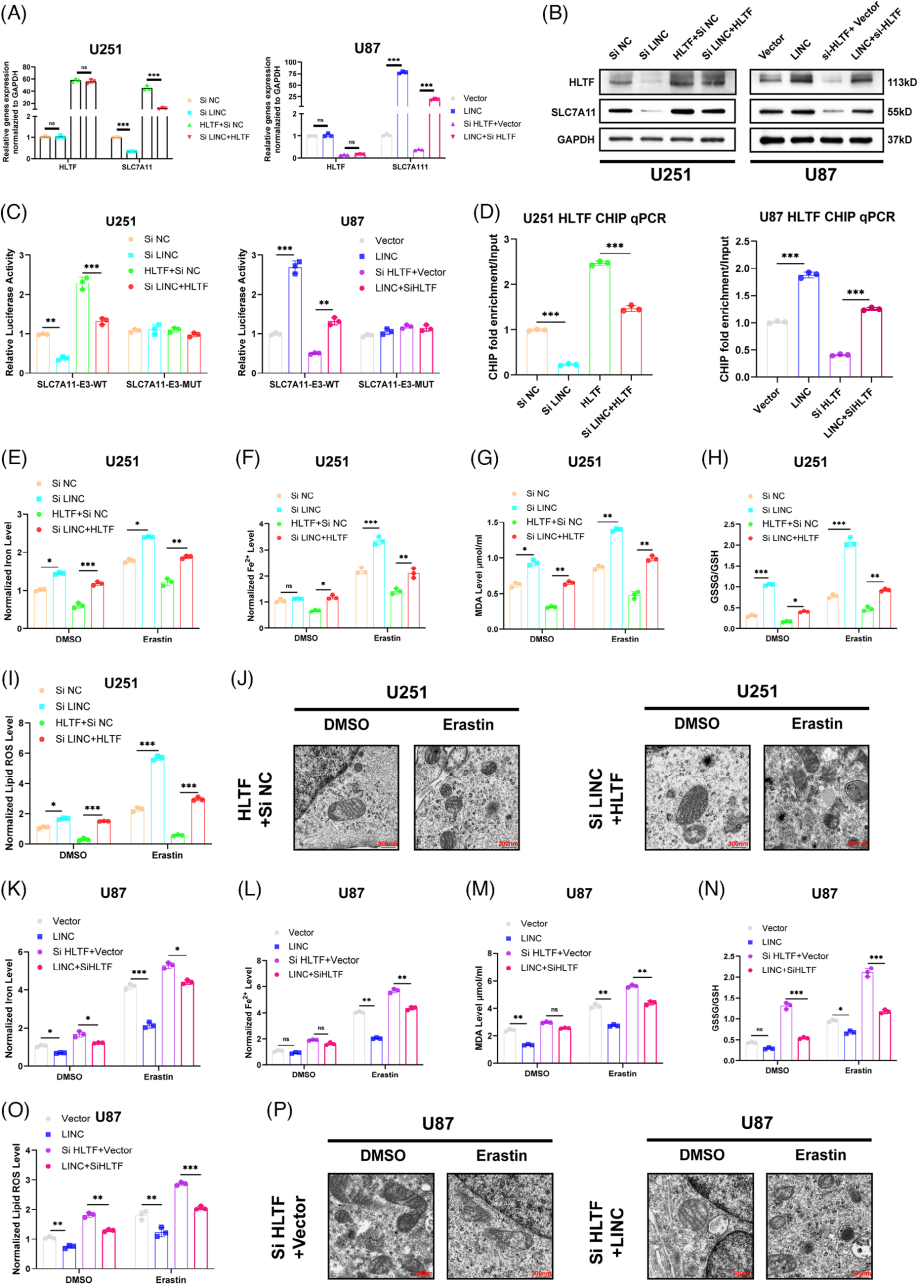
LINC01088 with binding to HLTF up‐regulates SLC7A11 transcription and inhibits ferroptosis in GBM. (A)b: qRT‐PCR was used to detect the relative expression of SLC7A11 and HLTF after rescue experiments in U251 and U87MG cell lines. (B) The protein expression of SLC7A11 and HLTF after rescue experiments in U251 and U87MG cell lines. (C) After the wild‐type plasmid or mutant plasmid is co‐transfected with the required plasmid or RNAi for the rescue experiments, and fluorescence intensity was detected in U251and U87MG cell lines. (D) The enrichment of HLTF antibody and promoter binding sites under different recovery conditions was analysed by ChIP‐qPCR. (E–J) The corresponding rescue experiments was performed in U251 cell line and determined Fe (E), Fe^2+^ (F), MDA(G), GSSGto GSH ratio (H), lipid ROS (I), and mitochondrial morphology was observed by transmission electron microscopy (J). (K–P) The corresponding rescue experiments was performed in U251 cell line and determined Fe (K), Fe^2+^ (L), MDA (M), GSSGto GSH ratio (N), lipid ROS (O), and mitochondrial morphology was observed by transmission electron microscopy (P). *p < 0.05, **p < 0.01, ***p < 0.001, ****p < 0.0001, ns: not significant.

Rescue experiments were performed to investigate whether the interaction between LINC01088 and HLTF resulted in the upregulation of SLC7A11 transcription and subsequent inhibition of ferroptosis in GBM cells. The results demonstrated that overexpression of HLTF partially rescued the inhibitory effect of LINC01088 knockdown on SLC7A11 expression at both the mRNA and protein levels. Conversely, knockdown of HLTF attenuated the promoting effect of LINC01088 on SLC7A11 expression (Figure 7A,B). At the molecular level, the overexpression of HLTF partially restored the downregulation of SLC7A11‐E3‐WT caused by LINC01088 knockdown and reduced the enrichment of the HLTF antibody in the promoter region. Conversely, knockdown of HLTF weakened the upregulation of SLC7A11‐E3‐WT induced by LINC01088 and enhanced enrichment of HLTF antibody in the promoter region (Figure 7C,D). In terms of cellular function, the overexpression of HLTF partially rescued the promoting effect of LINC01088 knockdown on ferroptosis in GBM cells (Figure 7E–I), whereas knockdown of HLTF appeared to attenuate the inhibitory effect of LINC01088 on ferroptosis in GBM cells (Figure 7K–O). This was evident in the levels of total iron (Fe), ferrous iron (Fe^2+^), lipid ROS, the GSSG to GSH ratio, and MDA in GBM cells. An ultrastructural analysis using TEM further supported these findings (Figure 7J,P). Overall, these results further confirmed that the interaction between LINC01088 and HLTF leads to the upregulation of SLC7A11 transcription, consequently inhibiting ferroptosis in GBM cells.

### 3.8 Effects of LINC01088 on GBM tumour growth in vivo

**FIGURE 8:**
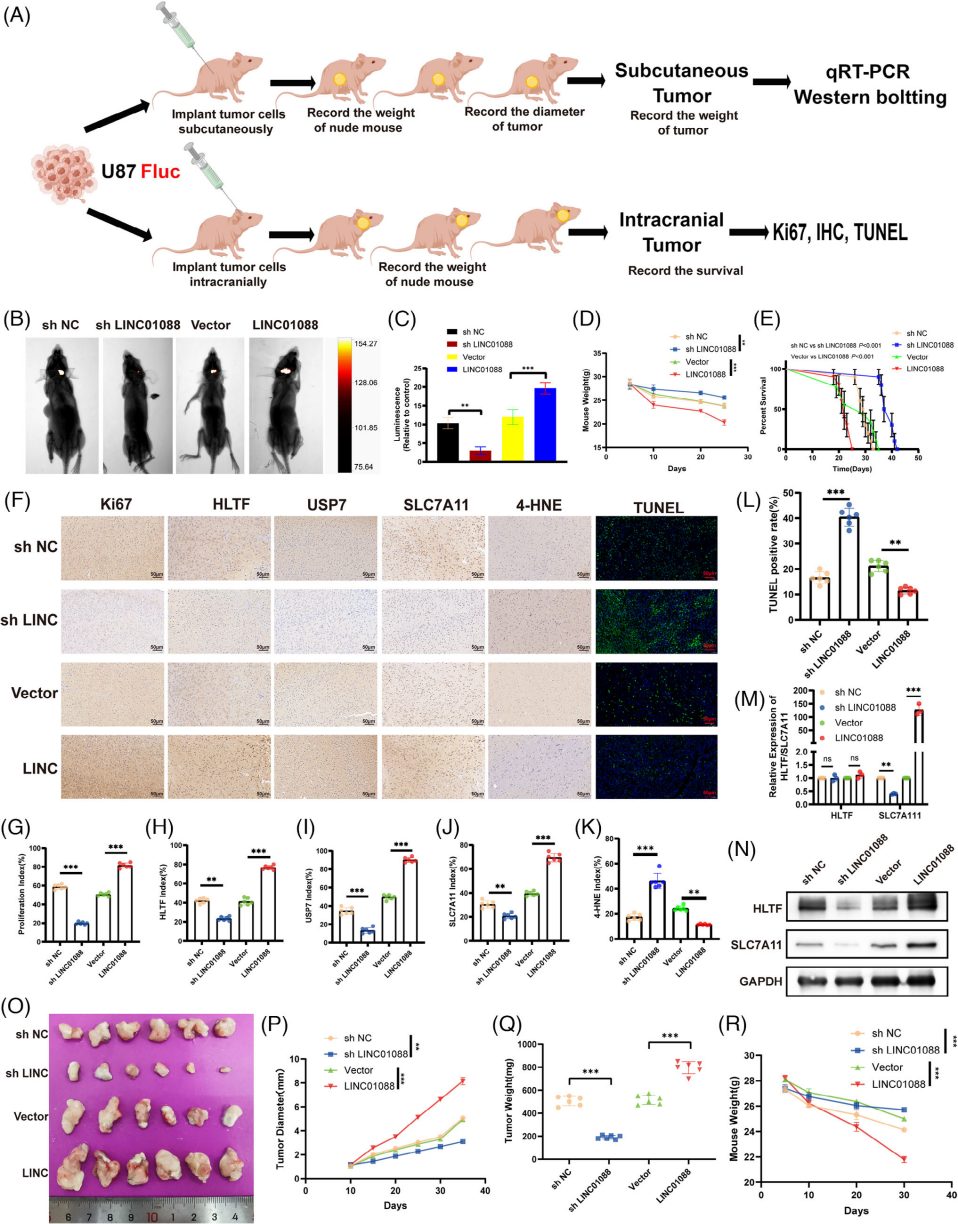
Effects of LINC01088 on GBM tumour growth in vivo. (A) Schematic illustration showing the design of animal experiments. (B) Representative live imaging images of nude mice in different experimental groups. (C) Tumour fluorescence intensity in different experimental groups. (D) Weight of nude mice in different experimental groups. (E) Survival curves of nude mice in different groups. (F–K) Representative charts of each immunohistochemistry (F), TUNEL staining and corresponding quantitative histogram, Ki67 (G), HLTF (H), USP7 (I), SLC7A11 (J) and TUNEL staining (K). (L) Representative picture of subcutaneous graft tumour. (M) Tumour diameter of subcutaneous graft tumours in different experimental groups. (N) Tumour weight of subcutaneous transplanted tumours in different experimental groups. (O) The decreased weight of mice in different experimental groups. (P) The mRNA relative expression levels of HLTF and SLC7A11 in subcutaneous transplanted tumour tissues were detected by qRT‐PCR. (Q) WB was used to detect the protein levels of HLTF and SLC7A11 in subcutaneous transplanted tumour tissues in different experimental groups. *p < 0.05, **p < 0.01, ***p < 0.001, ****p < 0.0001, ns: not significant.

To investigate the effects of LINC01088 on GBM in vivo, we employed both intracranial and subcutaneous xenograft models (Figure 8A). Notably, knockdown of LINC01088 led to reduced tumour growth and improved survival in the LINC01088‐SH group compared to those in the LINC01088‐NC group. Conversely, LINC01088‐OE promoted tumour growth and reduced survival (Figure 8B–E). Immunohistochemistry and TUNEL staining revealed that LINC01088‐SH reduced the expression of proteins such as Ki‐67, HLTF, USP7, and SLC7A11, thereby increasing GBM cell death. LINC01088‐OE had the opposite effect, elevating the expression of these proteins and suppressing cell death (Figure 8F–K). These results were consistent with our in vitro findings. The subcutaneous model paralleled the intracranial results (Figure 8L–O), and mRNA/protein profiles of the subcutaneous tumours matched the in vitro data (Figure 8P,Q).

## 4 DISCUSSION

The malignant transformation of tumour cells is a multistep process characterized by the accumulation of genetic mutations, chromosomal alterations, and epigenetic changes.^32^ LncRNAs are emerging as crucial regulatory factors in cancer biology, with 79% of them remaining unannotated.^33^ Previous cancer‐related studies have primarily focused on the relationship between protein‐coding gene mutations and cancer. However, lncRNAs have been widely reported to play regulatory roles in various biological processes in cancers, including resistance to cell death.^13^, ^14^ Ferroptosis is a novel area of cell death that has recently attracted considerable attention. Unlike other cell death mechanisms, ferroptosis is characterized by iron‐dependent lipid peroxidation accumulation, ultimately leading to cell death. Ferroptosis involves multiple pathways, including iron, glutathione, and lipid metabolism, each with specific molecular cascades and regulatory mechanisms.^34^ Although the key signalling pathways of ferroptosis have gradually been elucidated, the role and mechanisms of lncRNAs in ferroptosis remain unclear and warrant further exploration. Over the past decade, many stable lncRNAs have been identified in the bloodstream, and circulating lncRNAs have proven to be effective molecular biomarkers in various cancer and non‐cancer disease prognosis models.^10^ Therefore, research on the relationship between lncRNAs and cancer holds the potential to provide new insights into cancer development, improve cancer diagnosis and treatment, identify new drug targets, and enhance the long‐term quality of life of patients.

LINC01088 is significantly overexpressed in GBM plays a regulatory role in various cancers. For instance, LINC01088 modulates the miR‐95/LATS2 pathway through a ceRNA mechanism, inhibiting tumour cell growth.^24^ LINC01088 directly targets miR‐548b‐5p and miR‐548c‐5p, promoting the expression of G3BP1 and PD‐L1, thereby driving colorectal cancer progression and immune evasion.^35^ LINC01088 suppresses p21 and promotes cell proliferation by interacting with EZH2 in non‐small cell lung cancer.^26^ These previous studies indicate that LINC01088 plays a critical role in tumour initiation and progression. However, their role in GBM have not been explored. In this study, we used dual‐luciferase reporter assays to demonstrate that LINC01088 is primarily regulated at the transcriptional level, with SP1 identified as the transcription factor regulating LINC01088. To investigate the function of LINC01088, we constructed stable GBM cell lines by LINC01088 knockdown or overexpression. The results showed that overexpression of LINC01088 significantly enhanced the proliferation, migration, and invasion capabilities of GBM cell lines U87MG and U251 and inhibited intracellular ferroptosis, whereas knockdown had the opposite effect.

LncRNAs have been shown to act as molecular scaffolds, forming interaction platforms with proteins, thereby regulating protein activity, transport, and stability. Our results suggest that LINC01088 can bind to HLTF and enhance HLTF protein stability by inhibiting ubiquitin‐proteasome degradation. HLTF^36^ is a 113  kDa protein‐encoding gene belonging to the SWI/SNF protein family and is known for its involvement in chromatin remodelling.^37^ The primary role of HLTF in genome stability was described as a ubiquitin E3 ligase that protects against UV sensitivity by polyubiquitinating PCNA.^38^ Later, in a broader replication stress context, HLTF were found to have reversal activity, such as in response to nucleotide depletion. HLTF are also known to silence genes through promoter hypermethylation or alternative mRNA splicing in various tumours, leading to the truncation of proteins lacking DNA repair domains.^39^ In this study, we demonstrated that LINC01088 enhances HLTF protein stability by inhibiting ubiquitin‐proteasome degradation, and USP7 was identified as the deubiquitinase responsible for stabilizing HLTF. Ubiquitination is a strictly regulated and critical post‐translational modification that can activate or deactivate oncogenic pathways.^40^, ^41^ Deubiquitinases remove single ubiquitin or polyubiquitin chains from substrate proteins, thereby stabilising target proteins. USP7 is a member of the USP family that is closely associated with cancer progression.^42^ In this study, USP7 was found to participate in the LINC01088‐mediated inhibition of ubiquitin‐proteasome degradation of HLTF, thereby enhancing HLTF expression in GBM cells. We further confirmed the binding of HLTF to the promoter region of SLC7A11, which promoted its transcription. SLC7A11, a cystine/glutamate reverse transporter, is a key regulator of ferroptosis.^43^ SLC7A11 downregulation can indirectly inhibit GPX4 activity, leading to lipid peroxidation accumulation, and ultimately inducing apoptosis.^44^ In recent years, SLC7A11 was found to be highly expressed in various solid tumours, including ovarian cancer and GBM, and is closely associated with treatment resistance.^44^ SLC7A11 is a specific amino acid transporter that plays a critical role in the regulation of apoptosis. The downregulation of SLC7A11 increased ROS levels, decreased GSH levels, induced cell death, and reduced sensitivity to Temozolomide in U251 glioblastoma cells. Additionally, ATF4 overexpression in U87MG and U251 cells has been found to promote cell proliferation and inhibit apoptosis by increasing SLC7A11 expression.^36^ Several studies have shown that p53 activation can bind to the SLC7A11 gene promoter, inhibiting SLC7A11 transcription and affecting GSH synthesis, thereby inducing ferroptosis.^45^ The knockdown of SLC7A11 in GBM cells or Nutlin‐3a‐induced p53 further negatively regulates SLC7A11, increasing ALOXE3 activity, promoting apoptosis, and inhibiting the growth and migration of in situ tumours in mice.^46^ The regulatory axis SP1‐LINC01088‐HLTF/USP7‐SLC7A11 identified in our study highlights a critical pathway through which ferroptosis resistance is mediated in glioblastoma (GBM). While targeting LINC01088 itself may offer a direct approach to disrupt this axis, our data suggest that HLTF stabilization via USP7 represents the most promising therapeutic node for intervention. Here, we elaborate on the rationale for prioritizing HLTF modulation and its implications for adjuvant therapy optimization. HLTF, a chromatin‐remodelling factor stabilized by LINC01088 through its interaction with USP7, directly activates the transcription of SLC7A11, a key suppressor of ferroptosis.^47^, ^48^ Genetic or pharmacological inhibition of HLTF significantly reduces SLC7A11 expression, thereby sensitizing GBM cells to ferroptosis in vitro and in vivo. This positions HLTF as a linchpin connecting upstream regulator downstream ferroptosis resistance. USP7, a deubiquitinase that stabilizes HLTF, offers a druggable interface.^49^ Small‐molecule inhibitors of USP7 have shown efficacy in destabilizing HLTF and downregulating SLC7A11 in preclinical models.^49^ This approach avoids the challenges of RNA‐targeted therapies (e.g., delivery barriers for LINC01088‐specific siRNAs) and may synergize with existing therapies. Radiation‐induced lipid peroxidation and chemotherapy‐triggered ROS accumulation could be amplified by HLTF inhibition, leading to ferroptosis‐dependent tumour cell death.^17^, ^21^ In summary, this study reveals a new regulatory mechanism in GBM by elucidating how LINC01088 inhibits apoptosis in GBM cells.

## 5 CONCLUSIONS

**FIGURE 9:**
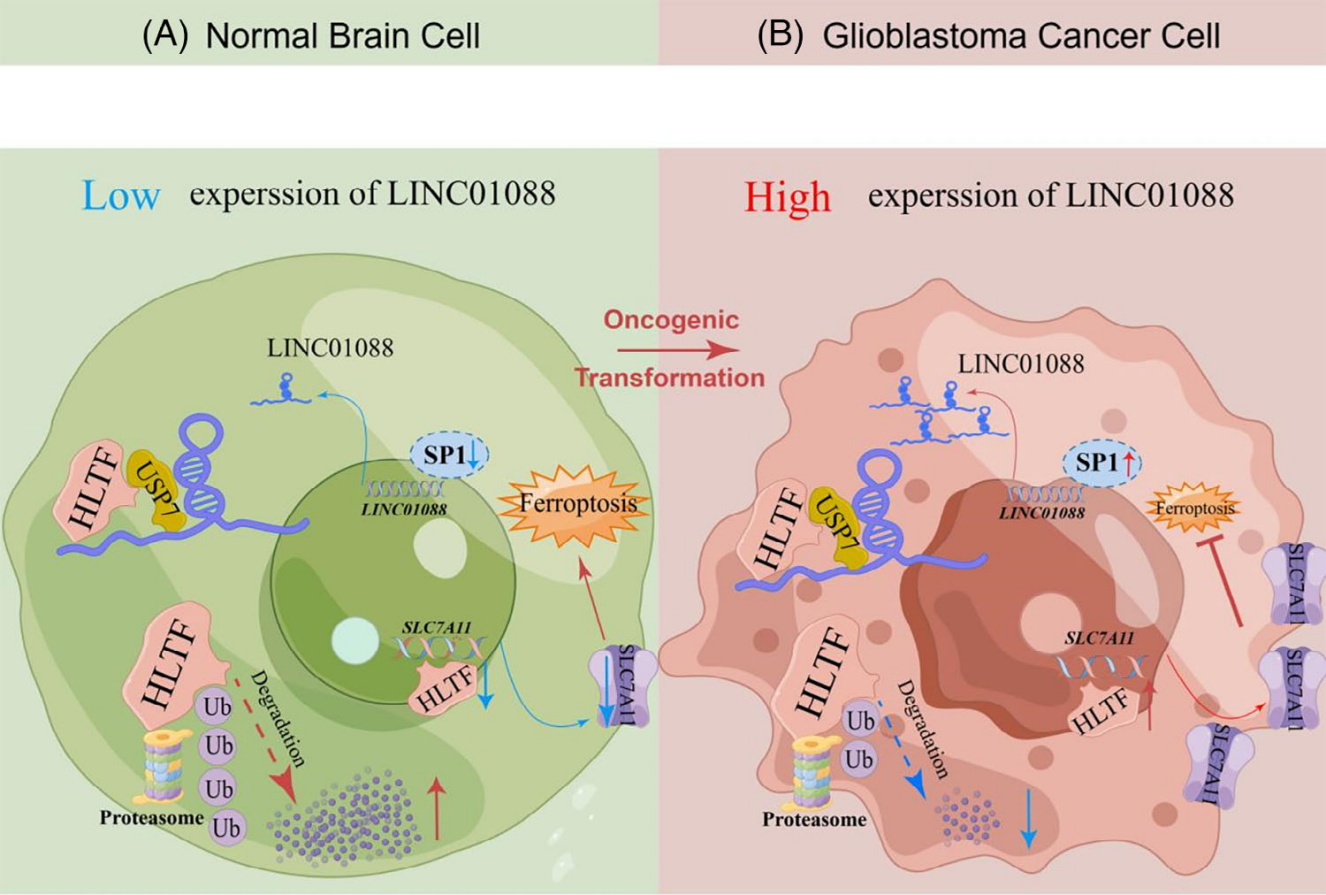
Schematic model detailing the molecular mechanisms of LINC01088 in normal brain cells and GBM cells. LINC01088 is transcriptionally upregulated by SP1. LINC01088 acts as a scaffold platform to bind USP7 and HLTF. USP7, as a deubiquitinating enzyme of HLTF, participates in inhibiting the ubiquitin‐proteasome degradation of HLTF, and HLTF further transcriptionally upregates the expression of downstream SLC7A11. Finally, ferroptosis of GBM cells was inhibited.

This study elucidates that LINC01088 functions as a scaffold platform to promote interactions between USP7 and HLTF. USP7 prevents HLTF degradation via the ubiquitin‐proteasome pathway to upregulate the downstream expression of SLC7A11, ultimately inhibiting ferroptosis in GBM cells (Figure 9). These findings reveal a novel regulatory pathway, positioning LINC01088 as a potential therapeutic target for ferroptosis‐based treatments in GBM.

## AUTHOR CONTRIBUTIONS

Xiaobing Jiang, Xuan Wang and Jianglin Zheng constructed this study. Yujie Zhou, Zhen Zhao and Cheng Jiang performed the experiments. Yujie Zhou, Zhipeng Wu, Hao Yu, and Dondong Xiao analysed the related data. Yujie Zhou and Zhen Zhao wrote the manuscript. Xiangbing Jiang, Jianglin Zheng and Xuan Wang were responsible for reviewing the manuscript and proposing the modifications in details. All authors contributed to this study and approved the submitted manuscript.

## CONFLICT OF INTEREST STATEMENT

The authors declare no conflicts of interest.

## ETHICS STATEMENT

Animal experiments were approved by the Committee on Ethics of Animal Experiments of Huazhong University of Science and Technology. Patient tissue sample collection was approved by the Institutional Review Board of Tongji Medical College at the Huazhong University of Science and Technology.

## CONSENT FOR PUBLICATION

All authors agree with the manuscript content.

## Supporting information



Supporting Information

Supporting Information

Supporting Information

Supporting Information

Supporting Information

Supporting Information

## Data Availability

The used and analysed datasets during this study are available from the corresponding author on reasonable request.
