# Supplementary material for: LINC01088 prevents ferroptosis in glioblastoma by enhancing SLC7A11 via HLTF/USP7 axis
Source: Clin Transl Med. 2025 Feb 25;15(3):e70257. doi: 10.1002/ctm2.70257 (PMC11859122; doi:10.1002/ctm2.70257)
Supplement: Supplementary file 1 — Supporting Information [file CTM2-15-e70257-s005.docx]

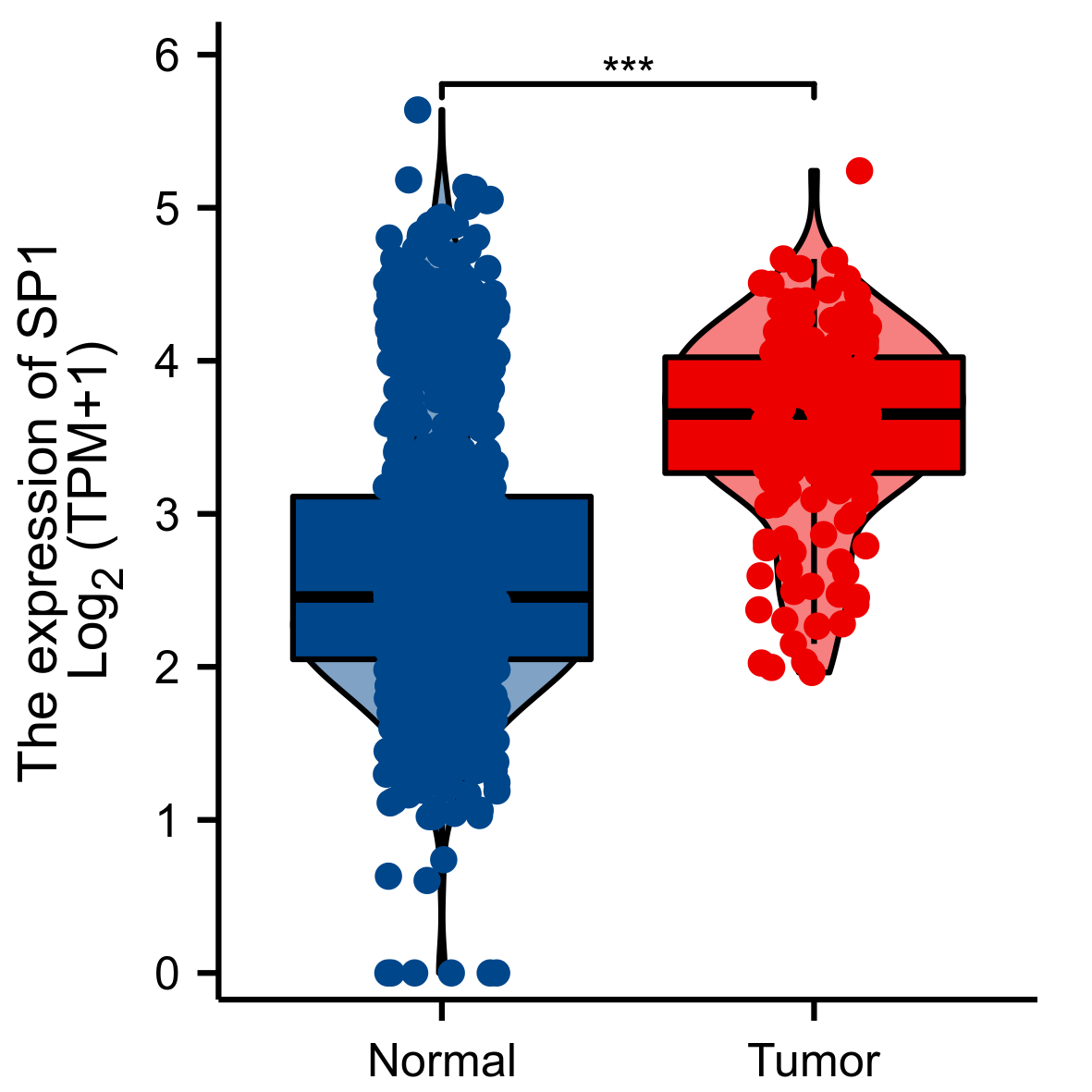


**Figure S1.** Relative mRNA expression levels of SP1 in Normal and Tumor in TCGA-GBM database. ***P < 0.001


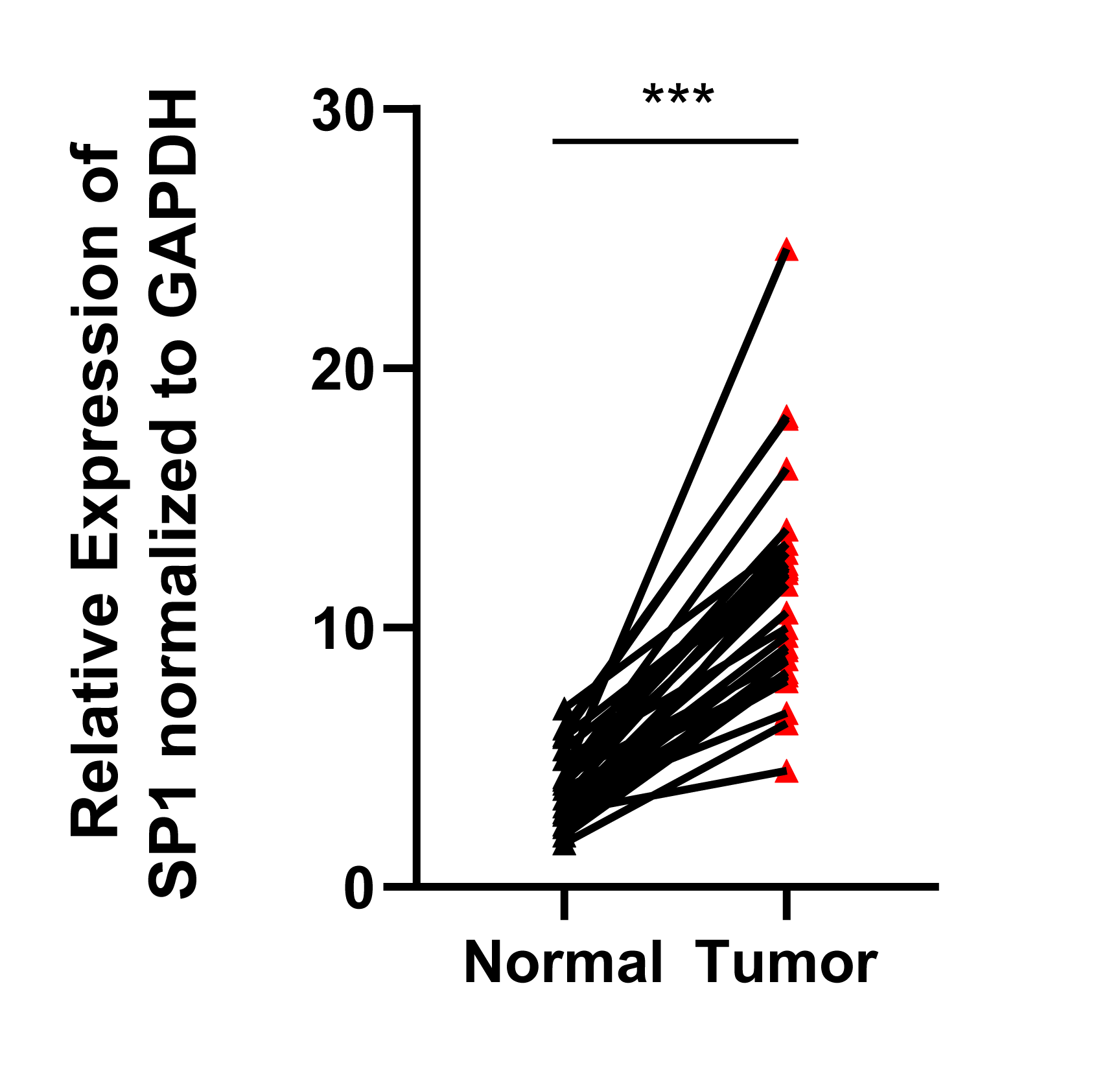


**Figure S2.** Relative mRNA expression levels of SP1 in Normal and Tumor tissue samples. ***P < 0.001


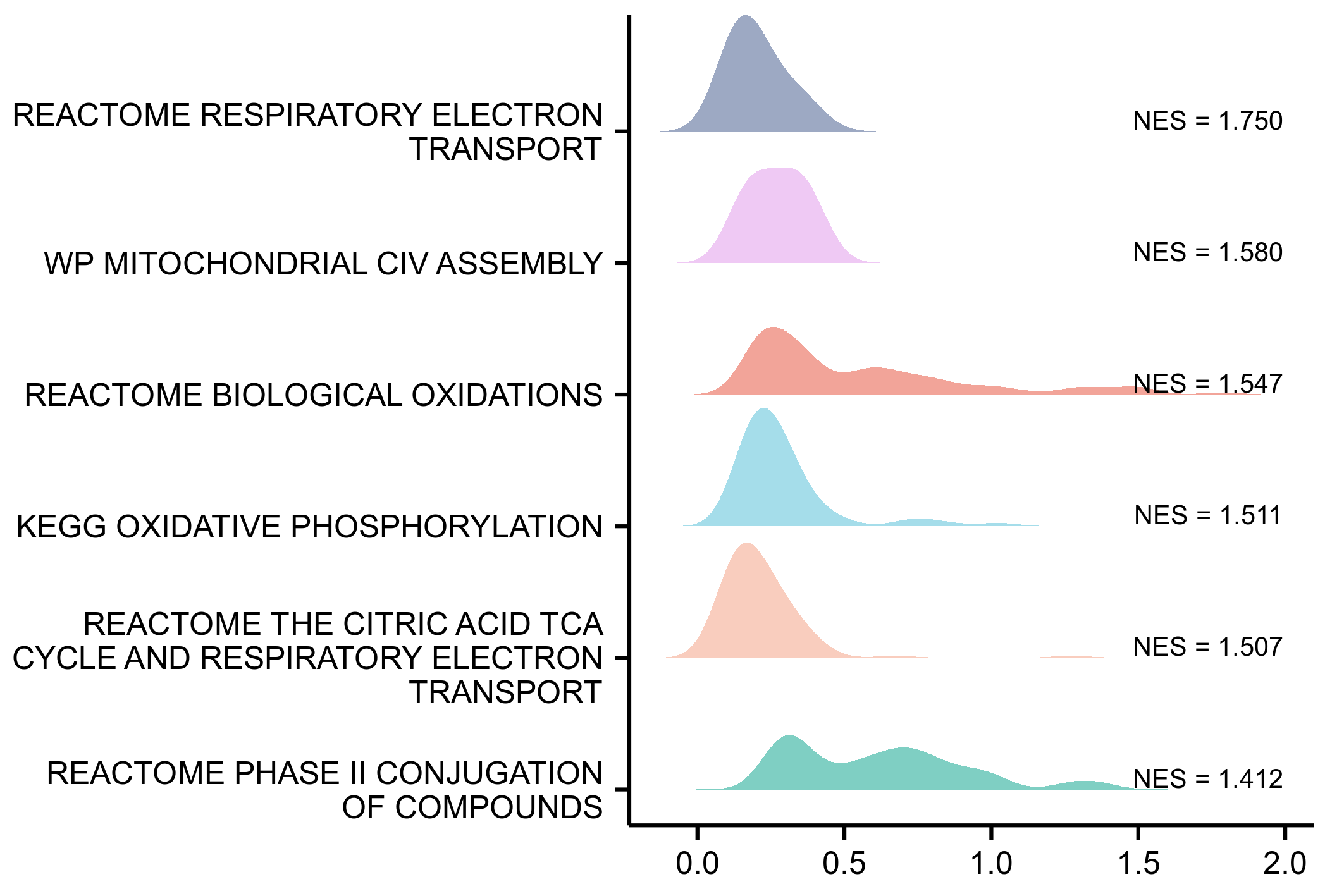


**Figure S3.** GSEA analysis of LINC01088 found that differential genes were mainly enriched in oxidative stress pathways


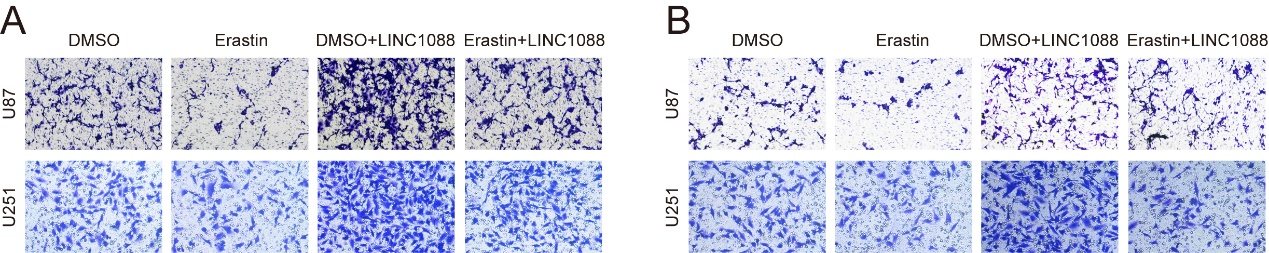


**Figure S4.** The trend of LINC01088-OE enhancing the GBM malignant progression can be offset by Erastin. A The transwell assays showed the migration abilities of U87 cells and U251 cells. B The transwell assays showed the invasion abilities of U87 cells and U251 cells.


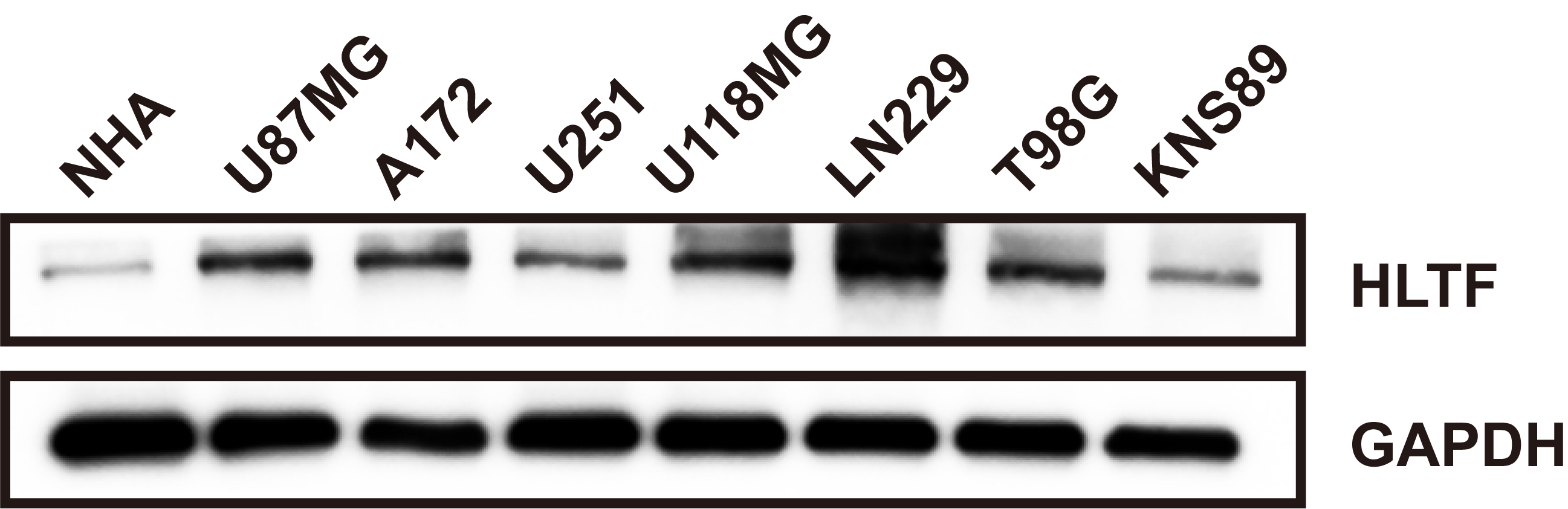


**Figure S5.** HLTF protein expression levels in NHA and GBM cell lines detected by western blot.


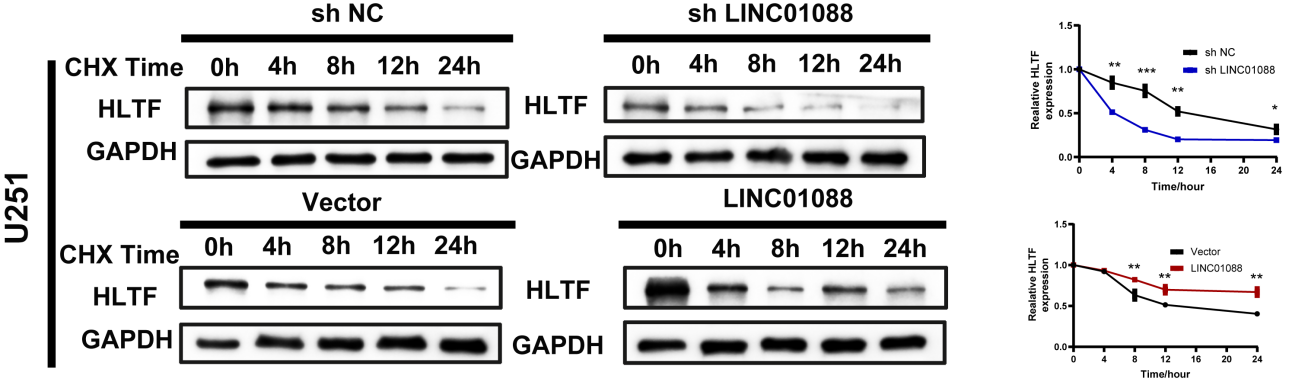


**Figure S6.** The protein expression level of HLTF was detected by WB after 60 μg/ml CHX treatment in U251 cells. ** P < 0.01, *** P < 0.001


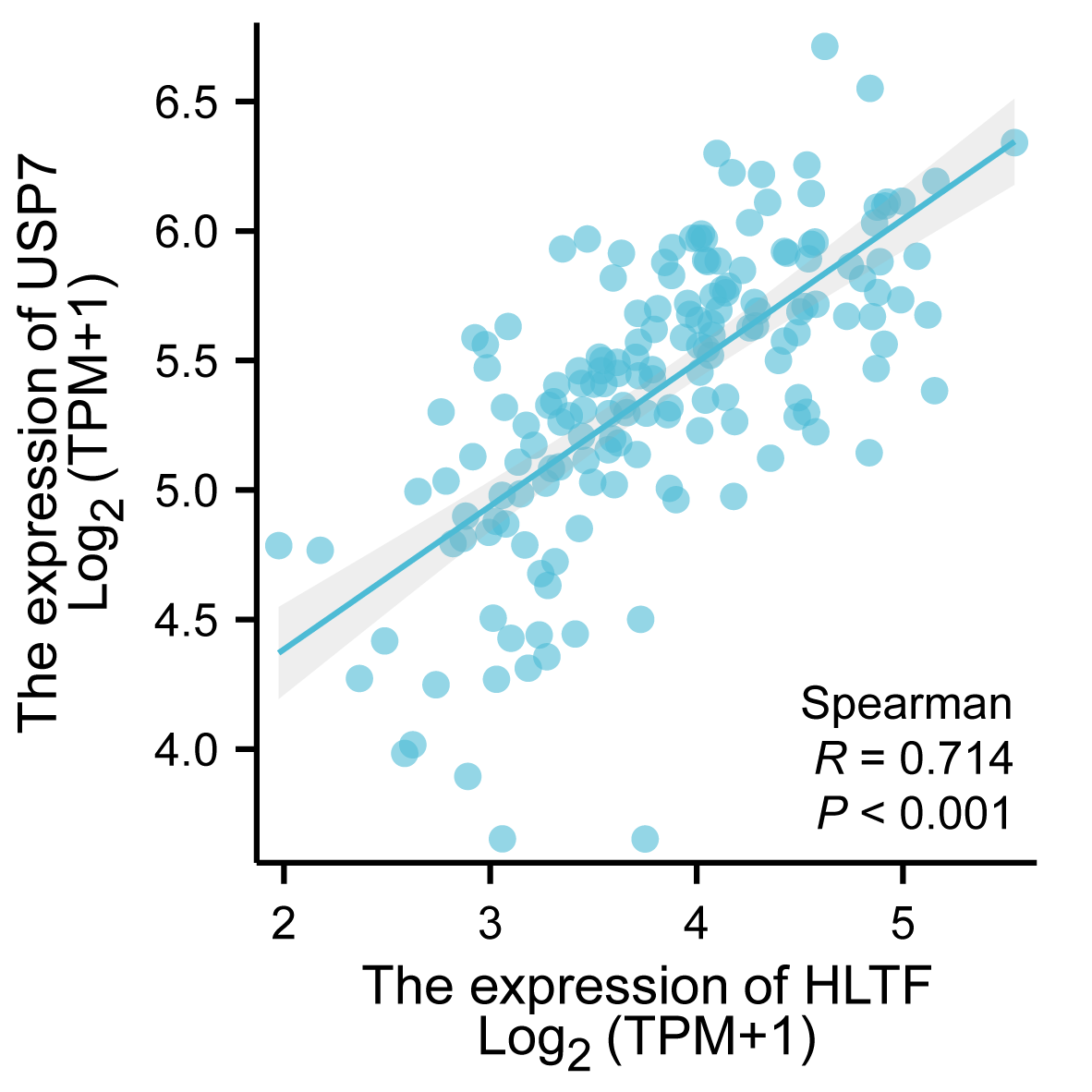


**Figure S7.** Correlation analysis of HLTF and USP7 genes in TCGA-GBM database.


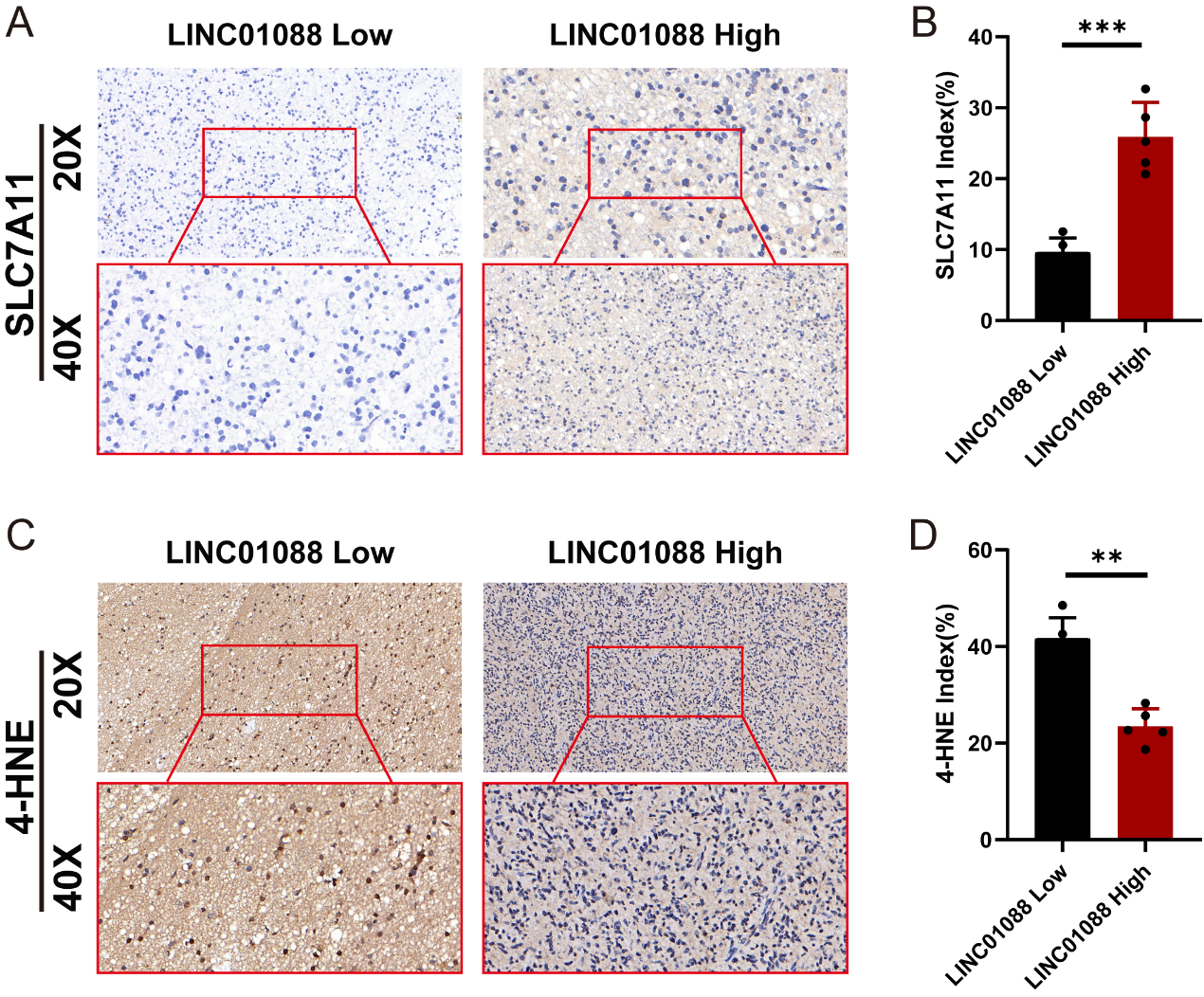


**Figure S8**. Correlation analysis of LINC01088 expression and SLC7A11 expression and 4-HNE expression in GBM specimen. A, B The expression of LINC01088 is positively correlated with SLC7A11 expression in GBM specimens(A), quantitative analysis of IHC (B). C, D The expression of LINC01088 is negatively correlated with iron death metabolite 4-HNE in GBM specimens (C), quantitative analysis of IHC (D).
