# Supplementary material for: LINC01088 prevents ferroptosis in glioblastoma by enhancing SLC7A11 via HLTF/USP7 axis
Source: Clin Transl Med. 2025 Feb 25;15(3):e70257. doi: 10.1002/ctm2.70257 (PMC11859122; doi:10.1002/ctm2.70257)
Supplement: Supplementary file 2 — Supporting Information [file CTM2-15-e70257-s006.docx]

**Supplementary Table 1. Clinical information of patients with Glioblastoma**

| **No.** | **Age** | **Gender** | **Pathological diagnosis** | **WHO grade** |
| --- | --- | --- | --- | --- |
| 1 | 28 | Male | Glioblastoma | 4 |
| 2 | 45 | Female | Glioblastoma | 4 |
| 3 | 67 | Male | Glioblastoma | 4 |
| 4 | 53 | Male | Glioblastoma | 4 |
| 5 | 64 | Female | Glioblastoma | 4 |
| 6 | 55 | Male | Glioblastoma | 4 |
| 7 | 57 | Female | Glioblastoma | 4 |
| 8 | 57 | Male | Glioblastoma | 4 |
| 9 | 56 | Male | Glioblastoma | 4 |
| 10 | 45 | Female | Glioblastoma | 4 |
| 11 | 61 | Male | Glioblastoma | 4 |
| 12 | 62 | Male | Glioblastoma | 4 |
| 13 | 33 | Female | Glioblastoma | 4 |
| 14 | 48 | Male | Glioblastoma | 4 |
| 15 | 55 | Female | Glioblastoma | 4 |
| 16 | 48 | Male | Glioblastoma | 4 |
| 17 | 57 | Male | Glioblastoma | 4 |
| 18 | 56 | Female | Glioblastoma | 4 |
| 19 | 62 | Male | Glioblastoma | 4 |
| 20 | 44 | Male | Glioblastoma | 4 |
| 21 | 54 | Female | Glioblastoma | 4 |
| 22 | 61 | Male | Glioblastoma | 4 |
| 23 | 60 | Female | Glioblastoma | 4 |
| 24 | 71 | Male | Glioblastoma | 4 |
| 25 | 33 | Female | Glioblastoma | 4 |
| 26 | 45 | Male | Glioblastoma | 4 |
| 27 | 53 | Male | Glioblastoma | 4 |
| 28 | 69 | Female | Glioblastoma | 4 |
| 29 | 38 | Female | Glioblastoma | 4 |
| 30 | 44 | Male | Glioblastoma | 4 |

**Supplementary Table 2. Plasmid vector sequence and siRNA used in this study**

| **Name** | **Sequence (5’ - 3’)** | **Company** |
| --- | --- | --- |
| PGMLV-CMV-H_LINC01088-EF1-ZsGreen1-T2A-Puro | NR_038342.1 | Genomeditech |
| pLVX-IRES-HLTF-puro | NM_003071.4-3FALG | Genomeditech |
| sh NC (PGMLV-SC5) | TTCTCCGAACGTGTCACGT | Genomeditech |
| H_LINC01088-shRNA1(PGMLV-SC5) | GCTCAGCGTTTCACAGCTAAG | Genomeditech |
| H_LINC01088-shRNA2(PGMLV-SC5) | GCATAATTCCCGGAAGATTAC | Genomeditech |
| H_LINC01088-shRNA3(PGMLV-SC5) | GGCTGTAAATTTCGTTGAATG | Genomeditech |

**Supplementary Table 3. Primers for RT-qPCR**

| **Name** | **Forward primer (5’ - 3’)** | **Reverse primer (5’ - 3’)** |
| --- | --- | --- |
| H_LINC01088 | AGGTTCACATGCCTCAAGGA | CCAGGCACAGAGGAGGACTA |
| H_FOXD3-AS1 | TCTGGCCTCAGTGCTCATTC | ACCTGAGTGGTTTGGTTGGG |
| H_Sp1 | GCCCTCTGACCAAGATCACT | TGGGTGACTCAATTCTGCTGC |
| H_HLTF | TTTGGAAAGTGGTTGGGGCT | GAAGATCCTGCTTTCGCCCT |
| H_SLC7A11 | TGGAACGAGGAGGTGGAGAA | TGGTGGACACAACAGGCTTT |
| H_GAPDH | ATCCCATCACCATCTTCC | ATGACCCTTTTGGCTCCC |
| H_U6 | CTCGCTTCGGCAGCACA | AACGCTTCACGAATTTGCGT |

**Supplementary Table 4. LINC01088 promoter sequence**

| **LINC01088 promoter sequence (Dual-luciferase reporter)** |
| --- |
| WT: GCTAGCTGTTGAATAATTTCTTTTTTCTTTTTTTTTTTTTTTTGAGACGGAGTCTCGTTCTGTCACCCAGGCTGGAGTGCAGTGGCATTATCTAGGCTCACTGCACACTCCACCTCCTGGGTTCATGCCATTCTCCTGCCTCAGCCTCCCGAGTAGCTGGGACTACAGGCGCCTGCCACCAGGCCTGGCTAATTTTTTGTATTTTTACTCGAG  MUT: GCTAGCTGTTGAATAATTTCTTTTTTCTTTTTTTTTTTTTTTTGAGACGGAGTCTCGTTCTGTCACCCAGGCTGGAGTGCAGTGGCATTATCTAGGCTCACTGCACGTCTTGTTCTTTGGGTTCATGCCATTCTCCTGCCTCAGCCTCCCGAGTAGCTGGGACTACAGGCGCCTGCCACCAGGCCTGGCTAATTTTTTGTATTTTTACTCGAG |

**Supplementary Table 5. SLC7A11 promoter sequence**

| **SLC7A11 promoter sequence (Dual-luciferase reporter)** |
| --- |
| WT: GGTACCTGTCACGTGGGTTTGTTTTACAGATTACTTCATCACCCATATATTAAGCCTAGTACCGAATAGTTCCTTTTTCTGCTCCTCTCCCTCCTTCAACCCTCCATGCTCAAACAGACCCCAGTGTCTGTTGTTCCCTTTATGTTCATGAGTTCTCATCATTTATCTCTCACTTATAAGTGAGAACATGTAGTATTTGGTTTTCTCTCGAG  MUT: GGTACCTGTCACGTGGGTTTGTTTTACAGATTACTTCATCGTTTGCGCGCTAAGCCTAGTACCGAATAGTTCCTTTTTCTGCTCCTCTCCCTCCTTCAACCCTCCATGCTCAAACAGACCCCAGTGTCTGTTGTTCCCTTTATGTTCATGAGTTCTCATCATTTATCTCTCACTTATAAGTGAGAACATGTAGTATTTGGTTTTCTCTCGAG |
